# Perivascular cells function as key mediators of mechanical and structural changes in vascular capillaries

**DOI:** 10.1126/sciadv.adp3789

**Published:** 2025-01-10

**Authors:** Cristiane M. Franca, Maria Elisa Lima Verde, Alice Correa Silva-Sousa, Amin Mansoorifar, Avathamsa Athirasala, Ramesh Subbiah, Anthony Tahayeri, Mauricio Sousa, May Anny Fraga, Rahul M. Visalakshan, Aaron Doe, Keith Beadle, McKenna Finley, Emilios Dimitriadis, Jennifer Bays, Marina Uroz, Kenneth M. Yamada, Christopher Chen, Luiz E. Bertassoni

**Affiliations:** ^1^ Knight Cancer Precision Biofabrication Hub, Knight Cancer Institute, OHSU, Portland, OR 97201, USA.; ^2^ Cancer Early Detection Advanced Research Center (CEDAR), Knight Cancer Institute, OHSU, Portland, OR 97201, USA.; ^3^ Department of Oral Rehabilitation and Biosciences, School of Dentistry, OHSU, Portland, OR 97201, USA.; ^4^ Piracicaba Dental School, State University of Campinas (UNICAMP), Piracicaba, Sao Paulo, SP 13414-230, Brazil.; ^5^ Trans-NIH Shared Resource for Biomedical Engineering and Physical Science, NIBIB, NIH, Bethesda, MD 20892, USA.; ^6^ Biological Design Center, Department of Biomedical Engineering, Boston University, Boston, MA 02215, USA.; ^7^ Wyss Institute for Biologically Inspired Engineering, Harvard University, Boston, MA 02215, USA.; ^8^ Cell Biology Section, NIDCR, NIH, Bethesda, MD 20892, USA.; ^9^ Division of Oncological Sciences, School of Medicine, OHSU, Portland, OR 97201, USA.; ^10^ Department of Biomedical Engineering, School of Medicine, OHSU, Portland, OR 97201, USA.

## Abstract

A hallmark of chronic and inflammatory diseases is the formation of a fibrotic and stiff extracellular matrix (ECM), typically associated with abnormal, leaky microvascular capillaries. Mechanisms explaining how the microvasculature responds to ECM alterations remain unknown. Here, we used a microphysiological model of capillaries on a chip mimicking the characteristics of healthy or fibrotic collagen to test the hypothesis that perivascular cells mediate the response of vascular capillaries to mechanical and structural changes in the human ECM. Capillaries engineered in altered fibrotic collagen had abnormal migration of perivascular cells, reduced pericyte differentiation, increased leakage, and higher regulation of inflammatory/remodeling genes, all regulated via *NOTCH3*, a known mediator of endothelial-perivascular cell communication. Capillaries engineered either with endothelial cells alone or with perivascular cells silenced for *NOTCH3* expression showed a minimal response to ECM alterations. These findings reveal a previously unknown mechanism of vascular response to changes in the ECM in health and disease.

## Teaser

Perivascular cells regulate vascular function in response to changes in the ECM.

## INTRODUCTION

Maintenance of microvascular homeostasis is orchestrated through a dynamic interplay between endothelial cells (ECs) and perivascular cells (PCs) with the extracellular matrix (ECM) (*1*). Collagen type I is the most abundant protein in the human ECM (*2*, *3*), and both its microarchitecture and mechanics are crucial for maintaining tissue function and driving disease progression (*4*, *5*). For example, increased collagen stiffness and bundled architecture are hallmark features of an altered ECM (*6*, *7*) and are associated with aging, chronic inflammatory diseases, fibrosis, and a cancer-promoting stroma (*8*, *9*). Another hallmark of these conditions is the alteration in the structure and function of vascular capillaries, where vessels show increased leakiness, abnormal morphology, and dysregulated remodeling (*10*–*13*). Although much work has been done to understand the response of ECs and PCs to different biomaterials (*10*–*13*), the specific mechanisms regulating the response of vascular capillaries to the ECM alterations occurring in health and disease remain poorly understood. Therefore, the implications for understanding the interplay between matrix microarchitecture and mechanics with the human microvasculature are wide ranging.

PCs are known to stabilize the vasculature (*3*, *14*) and play a substantial role in regulating vessel permeability (*15*, *16*). Mural cells have also been implicated in events associated with tissue injury (*17*), inflammation (*18*), and cancer progression (*19*, *20*). These cells occupy a strategic position on the abluminal side of capillaries, where they share the basement membrane with the endothelium, and establish the communication between ECs and the ECM primarily via *NOTCH3* in a juxtacrine manner (*21*, *22*). Given the strategic position of PCs in the microvasculature, here, we hypothesize that these cells may be responsible for a structural mechanism of transmission of architectural changes and mechanical forces that sense and respond to the changes in collagen fibrils surrounding vascular capillaries. Addressing this hypothesis may explain how the microvasculature adapts to the gradual alterations in the ECM occurring in chronic and inflammatory diseases, such as cancer, diabetes, and fibrosis, which may have broad implications for therapeutic interventions, disease biology, and regenerative engineering.

To test this hypothesis, we used an organotypic model of PC-supported vascular capillaries in collagen type I (*15*, *23*) with distinct fibrillar microarchitectures that were achieved by varying the temperature of fibrillogenesis (*24*) without changing the collagen concentration. This resulted in a spectrum of collagen type I substrates ranging from a low-stiffness reticular (finer, more netlike) network, akin to a healthy ECM, to a stiffer bundled fibrillar mesh, mimicking an altered, more fibrotic tissue. Our results demonstrate that vascular abnormalities in response to collagen changes are minimal when endothelial capillaries are cultured without PCs. On the other hand, PCs mediate the formation of increasingly abnormal and leaky capillaries, marked by high expression of inflammation and remodeling genes in stiff bundled collagen. Our findings therefore point to a critical and previously unknown role for PCs in determining the response of the vasculature to a dysregulated ECM.

## RESULTS

### PCs regulate vascular morphology as a function of collagen stiffness and architecture

**Fig. 1.**
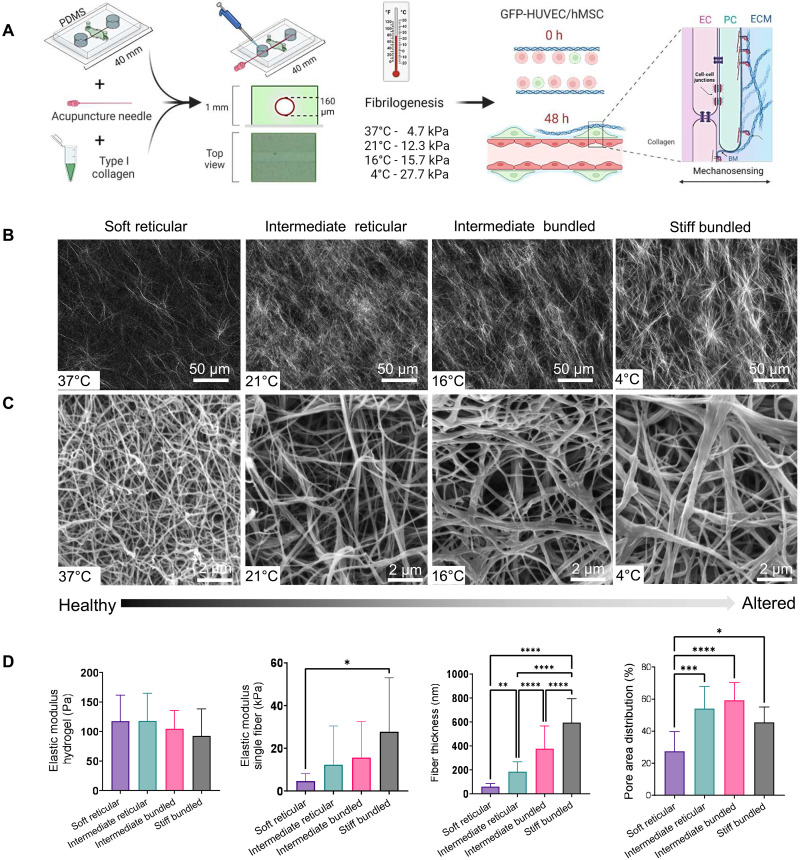
Device engineering and collagen characterization. (**A**) Schematic diagram showing the steps to engineer perivascularly supported capillaries on-a-chip with different collagen stiffness and microarchitectures. h, hours. (**B**) Collagen fibers became progressively thicker and more bundled with lower temperatures in a controllable manner as demonstrated by the second harmonic generation images. (**C**) Representative scanning electron microscopy images of collagen mesh after fibrillogenesis with different temperatures with gradual changes from soft reticular to stiff bundled. (**D**) Altering the fibrillogenesis temperatures did not change the bulk modulus but caused the average single collagen fibril elastic moduli to vary from 4.7 to 27.7 kPa, with progressive bundling of collagen fibers and heterogeneous pore distribution. *P < 0.05, **P < 0.01, ***P < 0.001, and ****P < 0.0001.

To investigate the regulatory role of PCs in forming vascular capillaries within collagen of varying stiffness and microarchitectures, we used a microfluidic device (*23*, *25*) composed of a single channel surrounded by collagen type I cast into a polydimethylsiloxane (PDMS) mold (Fig. 1A). To control collagen fibril stiffness and architecture, we adjusted the collagen gelation temperature, resulting in distinct fiber diameters and porosity, while keeping adhesion ligand density constant, as previously described (*24*, *26*–*29*). Specifically, we polymerized rat tail collagen hydrogels at 4°, 16°, 21°, and 37°C, where higher temperatures produced a finer and more compact reticular collagen network (Fig. 1, B and C), and lower temperatures yielded a more porous matrix with a bundled fibril microarchitecture (Fig. 1C and fig. S1).

Subsequently, we examined whether these structural differences correlated with fibril stiffness. When the hydrogel bulk modulus was measured via nanoindentation, no differences were found among groups (Fig. 1D); however, when we used a sharp, conically tipped probe (*24*) to determine the stiffness of single fibrils, the differences in elastic modulus were significant (Fig. 1D and fig. S2) and consistent with highly reticular fibers (37°C) having the lowest stiffness (4.7 kPa), and thicker fibrillar bundles (4°C) displaying significantly higher stiffness (27.7 kPa). Collagen assembled at 21° and 16°C displayed intermediate stiffness of 12.3 and 15.7 kPa, respectively, as illustrated in Fig. 1. Moreover, as temperatures decreased, we observed an increase in fiber thickness and pore area distribution (Fig. 1C and fig. S1).

**Fig. 2.**
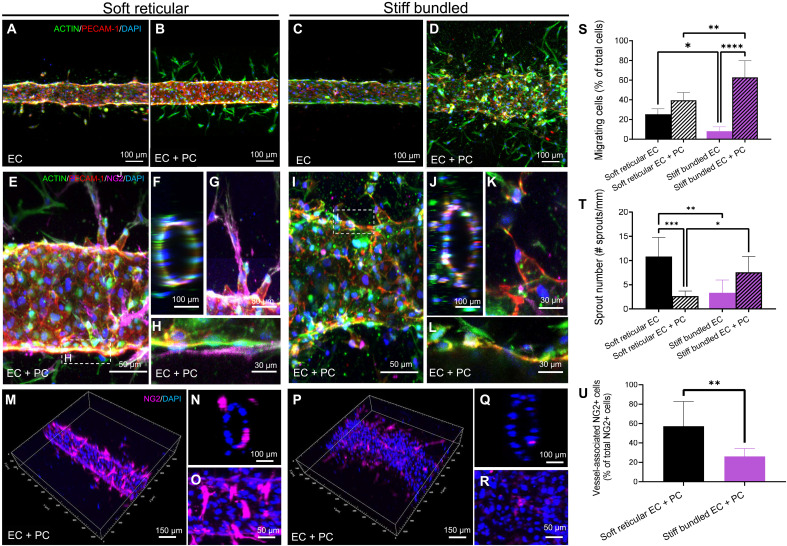
Vasculature characterization. (**A** and **C**) Engineered capillaries with only ECs presented similar morphologies irrespective of collagen stiffness and microarchitecture. The presence of PCs resulted in more disrupted morphology and abluminal migrating cells in stiff bundled collagen (D) and (**S**). In addition, the presence of PCs (**B** and **D**) markedly changed the morphology and number of capillaries within stiff bundled collagen, leading to larger and more irregular vessels than in soft reticular collagen. (**E** to **L** and **T**) Capillaries engineered in both soft reticular and stiff bundled collagen showed angiogenic sprouts; however, only those in soft reticular fibrils had pericyte coverage [(E) to (H)]. (**M** to **O**) Capillaries engineered in soft reticular collagen also showed abundant pericyte coverage, while vasculature within stiff bundled collagen had fewer NG2-positive cells associated with the endothelial wall (**P** to **R** and **U**). *P < 0.05, **P < 0.01, ***P < 0.001, and ****P < 0.0001.

We then examined the impact of collagen stiffness and microarchitecture on the formation of vascular capillaries by ECs cultured alone in the engineered microchannels (Fig. 2). We found that vessel formation occurred regardless of collagen stiffness, with minimal cell migration into the bulk collagen in all groups (Fig. 2, A and C), with exception of a reduction in the number of sprouts from soft reticular to stiff bundled collagen (fig. S3). Unexpectedly, capillary morphology remained virtually unchanged, irrespective of collagen stiffness and architecture.

Next, we coseeded human umbilical vein ECs (HUVECs) and human bone marrow mesenchymal stem cells (hMSCs) at a 4:1 ratio at a cell density of 8 × 10^6^ cells/ml in the microchannels. Within 24 hours, cells organized into an endothelialized capillary, consisting of an inner layer of ECs surrounded by an outer layer of hMSCs (Fig. 2, E to L), resembling the in vivo morphology of pericyte cells in the vasculature (Fig. 2, M to O). hMSCs within soft reticular collagen aligned longitudinally along the vessels and remained closely associated with the ECs (Fig. 2, E to H, M to O, and S). Stiff bundled collagen led to increasing migration and proliferation of PCs (Fig. 2, B and D) toward the abluminal side of the vascular ECM (Fig. 2, I to L, P to R, and S). In addition, vessel morphology underwent substantial changes as collagen became thicker in the stiff bundled group, with a significant increase in vascular diameter (fig. S3).

To assess whether collagen stiffness and microarchitecture influenced the differentiation of hMSCs into a pericyte-like phenotype (*2*), we quantified neuron-glial antigen 2 (NG2)–positive cells adhered to the vessel wall. Our findings showed a consistent inverse correlation between stiffness and NG2 expression across all groups we tested (Fig. 2, M to R, and figs. S4 and S5). Accordingly, soft reticular collagen promoted pericyte differentiation near the vessel walls (Fig. 2, E to H and U), while stiff bundled collagen had the opposite effect, showing the highest number of abluminal migrating cells and significantly fewer NG2-positive cells (Fig. 2, I to L, P to R, and U). The presence of PCs was also associated with a lower sprout length and number in soft reticular collagen, while stiff bundled fibrils had longer sprouts. This effect was consistently found across all stiffness ranges we tested (figs. S4 and S5). This was opposite to the effects found in capillaries engineered with ECs only (Fig. 2, T and U, and fig. S6).

### PCs regulate endothelial adhesion and barrier function in a stiffness- and architecture-dependent manner

**Fig. 3.**
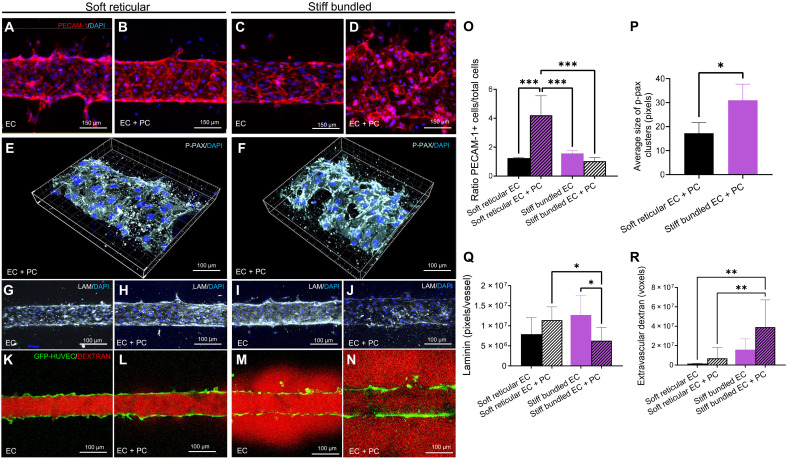
Cell-cell junctions, basement membrane, and barrier function of the vasculature. (**A** to **D**) Capillaries with only ECs expressed PECAM-1 at comparable levels, while PECAM-1 is increased in capillaries with PCs in soft reticular collagen. Heterogeneous and decreased expression of PECAM-1 is observed in capillaries engineered with PCs in stiff bundled collagen. (**E** and **F**) p-pax is overexpressed in vasculature engineered in stiff collagen. (**G** to **J**) Laminin (LAM) was expressed in both groups regardless of the absence of PCs; however, PCs in stiff bundled collagen showed irregular deposition of laminin across the capillary. (**K** to **N**) Vascular capillaries engineered in soft reticular collagen preserved the barrier function regardless of the presence of PCs, while stiff bundled collagen was associated with reduced barrier function even in the presence of PCs. *P < 0.05, **P < 0.01, and ***P < 0.001.

To characterize EC adhesion as a function of stiffness and architecture, we again engineered capillaries with and without PCs and compared soft reticular collagen versus stiff bundled fibrils in vessels stained for PECAM-1/CD31 (platelet and EC adhesion molecule) and N-cadherin (neural cadherin) (*30*). Pericytes induced a significant increase in endothelial PECAM-1 expression within the soft reticular collagen but not within the stiff bundled fibrils (Fig. 3, A to D and O, and fig. S7). In contrast, N-cadherin expression was prominently observed in both ECs and PCs in the stiff bundled group, while in the soft reticular group, it was predominantly confined to PCs. Notably, in the absence of PCs, ECs displayed limited N-cadherin expression (fig. S8) in stiff bundled collagen.

To further understand the ECM-vasculature interactions in soft reticular and stiff bundled collagen, we characterized the expression of paxillin, a key component of focal adhesions that plays a role in the transduction of extracellular signals into intracellular responses, triggered by the engagement of integrins with the ECM. Upon integrin engagement with the ECM, paxillin is phosphorylated, activating numerous signaling events associated with cell migration (*31*). While cells in the soft reticular collagen showed a sparse distribution of phosphorylated paxillin (p-pax), the stiff bundled collagen displayed enhanced p-pax that was concentrated adjacent to actin stress fibers (Fig. 3, E and F) and tended to concentrate on the cell membrane of PCs (fig. S9). Contrarily, laminin, a critical basement membrane component, was significantly reduced only in capillaries engineered using PCs within stiff bundled collagen (Fig. 3, G and J).

On the basis of our observations that stiff bundled collagen drives perivascularly induced changes in endothelial adhesion, we examined whether there was a change in vascular barrier function. Overall, variations in the anchorage and cell-cell adhesion systems led to sustained barrier function in vascular capillaries engineered with PCs in soft reticular collagen but disrupted the barrier function in capillaries engineered with PCs in stiff bundled collagen (Fig. 3, K to N). The presence of PCs maintained the barrier function even when collagen had an intermediate stiffness of ~12.3 kPa that was almost three times the stiffness of soft reticular collagen (~4.7 kPa). However, such a barrier function was not preserved when the stiffness was ~15.7 kPa, and the collagen mesh was arranged in bundles (fig. S10). The positive influence of a soft reticular ECM in endothelial cell-cell adhesion was only significant in the presence of PCs. Similarly, the negative impact of a stiff bundled architecture on cell-matrix interactions and barrier function was only observed when ECs were cocultured with PCs. Together, these data point to the critical role of PCs in mediating both structural and mechanical events in vascular capillaries.

### PCs in stiff bundled collagen modulate genes associated with inflammation and remodeling

**Fig. 4.**
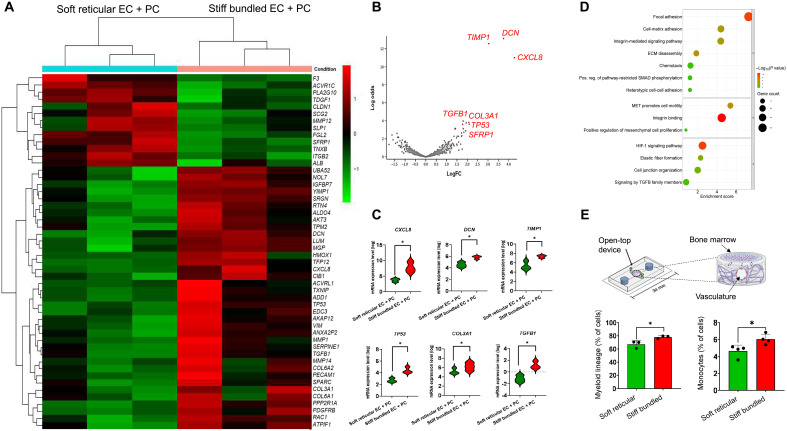
Gene analysis. (**A**) Heatmap comparing the effect of collagen stiffness and architecture on gene expression in the presence of PCs. (**B**) Volcano plot showing that genes encoding for *DCN*, *CXCL8*, and *TIMP1* were most highly expressed by the vasculature in a stiff environment. (**C**) Pairwise comparison of the nine most different log values for gene expression. (**D**) Enrichment score and pathways associated with capillaries engineered in either soft reticular or stiff bundled collagen. (**E**) Engineered bone marrow cells were seeded on top of the soft and stiff vasculature and allowed to interact for 3 days. In the presence of the vasculature in the stiff matrix, bone marrow cells tended to express more monocytic and myeloid markers consistent with IL-8 (inflammatory) stimulation.

To further understand the role of PCs in the morphological and functional differences observed between soft reticular and stiff bundled collagen, we screened 770 genes using a NanoString gene panel (dataset S1 and fig. S11). From the top 10 most differentially expressed genes in the vasculature engineered in stiff bundled collagen compared to that engineered in soft reticular collagen, we identified genes associated with inflammatory signaling (*CXCL8*), ECM synthesis and remodeling (*TP53*, *TIMP1*, *COL3A1*, *MMP1*, and *DCN*), cell proliferation and differentiation (*TGFB1*, *ACVR1C*, and *SFRP1*), and cell-cell junction (*CLDN*) (Fig. 4, A to C). Enrichment scores showed that capillaries engineered in stiff bundled collagen activated pathways related to cell migration, endothelial proliferation, lysil oxidase remodeling, chemotaxis, and cancer (Fig. 4D and figs. S11 to S15). On the other hand, capillaries in soft reticular fibrils activated pathways related to cell junction organization, transforming growth factor–β1 (TGFB1) signaling and control of chemotaxis, cell adhesion, among other signaling (Fig. 4D and figs. S11 to S16).

To dissect the specific role of PCs in a stiff bundled collagen matrix, we compared the expression data between capillaries with and without PCs (fig. S17). We found that PCs were particularly relevant in modulating genes related to the ECM such as *COL1A2*, *COL1A1*, *COL6A2*, *FN1*, *LOX*, *DCN*, *TIMP1*, and especially *CXCL8*. The presence of PCs in a soft reticular environment led to the increase in *TGFB1* gene expression and pathways related to cell adhesion, integrin binding, and ECM receptor interaction (fig. S16).

*CXCL8* was the most differentially expressed gene in the stiff bundled collagen when ECs were cocultured with PCs. Building upon the established connection of interleukin-8 (IL-8, coded by the *CXCL8* gene) and inflammatory cell recruitment, we sought to further determine the effect of a stiff bundled microenvironment in vascular inflammatory paracrine signaling and chemotaxis of inflammatory precursor cells from the bone marrow. To that end, we prepared vascular channels using the same method used throughout the study, but in open-top microdevices, and seeded an organotypic bone marrow model on top of the engineered capillaries (Fig. 4E). Cells in the engineered bone marrow had significantly higher differentiation into monocytic and myelocytic lineages in the presence of capillaries engineered in stiff bundled collagen and similar differentiation into neutrophils and hMSCs (figs. S18 and S19).

### Silencing *NOTCH3* restores pericyte coverage, capillary morphology, and barrier function

**Fig. 5.**
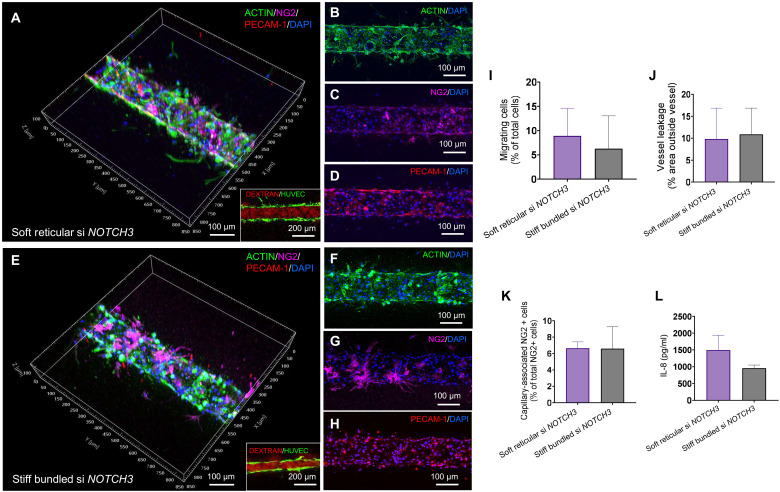
Normalization of vasculature after NOTCH3 silencing. Morphology (**A**, **B**, **E**, and **F**), pericyte coverage (**C** and **G**), and PECAM-1 expression (**D** and **H**) were similar between the vasculature engineered in a soft reticular or stiff bundled environment. (**I** to **K**) Migrating cells, pericyte coverage, and vessel leakage were around 10%, similar in both groups. (**L**) In addition, IL-8 secretion was comparable for both groups. Nonsilenced controls are shown in fig. S20.

Our results showed marked differences between soft reticular and stiff bundled groups when vessels were engineered with PCs. Moreover, our enrichment score analyses showed a notable effect of matrix adhesions and cell-matrix interactions (Fig. 4D). Therefore, we first targeted the interaction between PCs with the matrix by silencing the integrin B1 gene (*ITGB1*) in hMSCs before seeding the coculture into the collagen channels. We hypothesized that blocking integrins would normalize the vasculature in the stiff bundled collagen. However, contrary to our expectation, not only vessels engineered in stiff bundled collagen did not normalize, but also the soft reticular collagen showed increased abluminal cell migration and less pericyte coverage, thus pointing to the importance of establishing adequate PC-matrix interaction irrespective of the matrix characteristics (figs. S20 and S21). These results therefore suggested that our findings might be mediated at the cell-cell interface between PCs and ECs. Since *NOTCH3* has been demonstrated to be a key mediator of the interaction between ECs and PCs (*21*), we hypothesized that PCs may sense the structure and mechanics of the ECM and “convey” this information to underlying ECs via a *NOTCH3-*mediated mechanism of juxtacrine communication. To test that, we used gene silencing via small interfering RNA (siRNA) to specifically target *NOTCH3* in PCs. Our results showed that silencing of *NOTCH3* decreased PC migration significantly in stiff bundled capillaries, bringing it to levels that were statistically comparable to those of soft reticular collagen (Fig. 5, A, E, and I). A similar effect was observed for NG2 expression (Fig. 5, C, G, and K), indicating a rescue in the ability of these cells to differentiate into pericytes. Moreover, *NOTCH3* silencing significantly improved the vessel barrier function in the stiff bundled group to levels that were comparable to those observed within soft reticular collagen (Fig. 5, A, E, and J, insets). Since IL-8 (*CXCL8* gene) was the most highly expressed chemokine in the stiff bundled collagen group, we tested whether *NOTCH3* silencing resulted in a reduction in the secretion of IL-8 by the engineered vessels. As expected, our results confirmed that the reversal in vascular morphology and function observed upon *NOTCH3* silencing was reflected in IL-8 paracrine signaling as well (Fig. 5L). Lastly, since the *TGFB1* gene was overexpressed in the presence of PCs in both soft and stiff collagen groups, we also silenced *TGFB1* to probe its regulatory function in perivascular structural mechanosensing. Of note, silencing *TGFB1* also normalized pericyte coverage and the number of migrating cells in the fibrotic vasculature (figs. S20 and S21), although not as markedly as for *NOTCH3*. In addition, silencing *TGFB1* affected the capillaries engineered in the soft reticular group as well.

## DISCUSSION

Changes in the structure and mechanics of collagen are key hallmarks of aging (*32*, *33*), tissue remodeling (*34*), wound healing (*35*), and many pathological conditions, including several premalignant lesions, cancers (*36*, *37*) and chronic inflammatory diseases (*38*, *39*) (fig. S22). However, the response of vascular capillaries to these dynamic alterations in the collagen ECM remains poorly understood. Here, we demonstrate that PCs are key mediators of the ability of capillaries to respond to collagen architectural and mechanical changes associated with ECM alterations around the vasculature. In the absence of PCs, vascular morphology was not significantly altered, irrespective of changes in fibril thickness, stiffness, and porosity (Fig. 2, A and C). Capillaries with PCs, on the other hand, responded to a progressively more fibrotic collagen matrix with abnormal abluminal migration (Fig. 2, B and D) and lowered expression of endothelial adhesion markers (PECAM-1) (Fig. 3, A to D) and pericyte differentiation (NG2) (Fig. 2, M to R), becoming leakier (Fig. 3, K to N) and more abnormally anchored to the matrix (Fig. 3, E to J). Moreover, PCs mediated the up-regulation of inflammatory cytokines and matrix-remodeling proteins in stiff bundled collagen (Fig. 4, B and C), depending on their communication with ECs (Fig. 5, A and E). These results point to a possible feedback loop, where PCs mediate vascular inflammation and remodeling, thus further exacerbating the ECM changes associated with the ongoing fibrotic alterations in the matrix.

Stiff ECM is recognized to result from the thicker arrangement and density of individual collagen fibrils (*6*). Collagen matrices have a hierarchical structure that self-assembles through multiple steps, resulting in fibers of varying lengths and diameters, depending on environmental conditions such as protein concentration, pH, and temperature (*24*, *29*). Understanding how collagen fibers deform within the tissue is crucial to bridging the gap between tissue-scale biomechanics and cell-scale mechanobiology (*40*). At the nanometer scale, collagen molecules’ binding energy ensures a highly specific and stable structure, essential for protein-protein interactions (for example, fibronectin-collagen) and ligand-receptor interactions such as integrin-mediated cell adhesion. At the micrometer scale, as fibrils become larger in diameter, the resulting increase in pore size and decrease in fibril entanglement reduce the overall entanglement and cross-link density within the network, leading to lower bulk stiffness (*40*–*42*). Cells interact with the ECM by applying traction forces through cell-ECM adhesions, which are typically nanometer- to micrometer-sized protein complexes. These interactions predominantly occur at the level of single collagen fibers, the primary sites for mechanical engagement (*40*–*42*). Therefore, while larger-diameter collagen fibrils are intrinsically stiffer at the single-fibril level, as measured by atomic force microscopy, their assembly into a hydrogel network with larger pores may not immediately influence overall bulk stiffness. This complex interplay between energy and entropy at different scales explains the observed inverse relationship between fibril stiffness and the bulk stiffness of the collagen hydrogel.

It is challenging to decouple the biological effects of cells sensing single fiber stiffness and porosity since different migratory mechanisms may be in place. In the soft reticular collagen, cells sense more compliant collagen fibers with less overall porosity, so migration will require proteolytic digestion of the ECM to open gaps that will be filled with endothelial tip cells or PCs. The literature shows that for angiogenic sprouts to happen, ECs begin to sprout from neighboring intact vessels (*43*–*45*). Endothelial tip cells are formed through the action of proteolytic invadopodia, which degrade the vascular basement membrane of intact vessels at focal points, allowing cell penetration through areas of least resistance. These tip cells interact with collagen mainly via integrins, realign the ECM through tension, and use metalloproteinases for the degradation of collagen. The following cells connect through vascular endothelial cadherin (VE-cadherin) and tight junctions, actively move, and deposit the basement membrane along the cell-ECM interface. As a result, endothelial sprouts create a path of least resistance and deposit a substrate that guides collective cell movement (*43*, *46*). In the case of the fibrillar bundled collagen, PCs will sense the increased stiffness of the collagen fibers, have more traction force via focal adhesion kinases (Fig. 3 and fig. S9), and extend filopodia filling the voids of the increased porosity, enhancing their migratory behavior to move faster into the ECM, following the path of less resistance through the pores, in a mechanism that is less dependent on proteolysis and MMPs.

We found that the expected pattern of endothelial monolayer formation, surrounded by tightly adhered pericytes, was gradually disrupted as fibrils became stiffer, which is likely to result from the bundling of multiple fibrils and the consequent increase in fibril thickness (figs. S3 and S4) (*8*, *24*). Two events are likely to be associated with this phenomenon. First, stiff matrices have been shown to reduce the differentiation of hMSCs into pericytes in cocultures with ECs (*11*, *47*). Our analysis revealed that stem cell–related genes such as CD44 and vimentin are significantly higher in the stiff bundled samples (fig. S22) than in the soft reticular samples. This suggests that when hMSCs are cocultured with ECs in a soft reticular collagen environment, most hMSCs tend to differentiate into pericytes within 48 hours, covering the endothelium and expressing specific mural markers like NG2. In contrast, in a stiff bundled environment, fewer hMSCs undergo differentiation into pericytes while maintaining higher expression levels of CD44 and vimentin. Second, poorly differentiated pericytes preserved the more migratory phenotype of hMSCs and were more likely to penetrate the pore spaces in the collagen matrix, thus reducing the confinement of ECs to the engineered channels. That phenomenon was only present when PCs were used, since EC-only capillaries did not show the same pattern of abluminal cell migration despite the same characteristics of the collagen matrix.

Notably, both paracrine and juxtacrine signaling plays crucial roles in the reciprocal cross-talk between pericytes and ECs. Pericytes and ECs share the same basement membrane and interact through peg-socket junctional complexes, adhesion plaques, and gap junctions (*2*, *3*, *45*), which are examples of pericyte-EC juxtacrine signaling. In this study, vasculature engineered in a soft reticular collagen matrix showed up-regulation of the *TGFB1* gene (fig. S17), which is responsible for pericyte recruitment via paracrine signaling, thus suggesting some paracrine participation in the process of pericyte-EC response to matrix changes.

Since ECs and differentiated pericytes share the vascular basement membrane, we sought to determine whether capillaries engineered in soft reticular collagen had a different pattern of cell-cell and cell-matrix adhesion than capillaries in stiff bundled fibrils. Paxillin, a key component of integrin signaling, requires tyrosine phosphorylation for integrin-mediated cytoskeletal reorganization and is a marker of activated focal adhesion kinases (*31*). The notably more clustered pattern of p-pax and overall lower expression of laminin in stiff bundled collagen point to important alterations off the anchorage system displayed in a soft reticular ECM versus that of a stiff-bundled matrix (Fig. 3, E and F). Endothelial cell-cell adhesion is mediated by VE-cadherin, while EC-to-PC contact is mediated by N-cadherin and NOTCH3 (*48*). N-cadherin is induced in PCs in response to vascular injury in models of stiffening and proliferation (*49*), and both mediate the vascular barrier function together (*21*, *49*, *50*). Similar to the pattern of expression of PECAM-1, p-pax immunofluorescence revealed regions without considerable expression (Fig. 3, E and F), indicating possible gaps in the cell-cell and cell-matrix communication systems contributing to significantly leakier vessels in more fibrotic collagen. Likewise, the expression of N-cadherin followed a similar pattern of alterations in stiff bundled versus soft reticular collagen hydrogels.

Assessment of the 770 genes (fig. S11 and dataset S1) expressed in capillaries with and without PCs showed a marked increase in *CXCL8* expression, which encodes for IL-8, a canonical marker of inflammation, in both soft reticular and stiff bundled collagen. We hypothesized that high expression of *CXCL8* should lead to increased levels of IL-8 and, consequently, an inflammatory response mediated by immune cells. Covering capillaries engineered in stiff bundled collagen with an organotypic model of bone marrow resulted in a significant increase in differentiation of human stem cells into monocytes and myeloid lineage cells (Fig. 4E). This supports the conjecture that IL-8, among other cytokines produced by the capillaries in a stiff bundled matrix, may exacerbate the action of inflammatory cells in the perivascular microenvironment, such as monocytes, neutrophils, and dendritic cells, which derive from a myeloid lineage (*51*), and potentially T cells, a finding that may have important implications for cancer progression and metastasis.

A proinflammatory microenvironment has been well established to accelerate matrix remodeling (*52*). Consistent with that notion, in stiff or fibrotic collagen we also found that decorin (*DCN*), a matrix proteoglycan that is important for collagen fibril assembly (*53*), *COL3A*, which encodes for collagen production, and tissue inhibitor of matrix metalloproteinase (*TIMP1*), which regulates matrix degradation, were all significantly up-regulated together with *TP53*, which is known as the master regulator of vascular remodeling (Fig. 4, B and C) (*54*, *55*). These findings indicate that a fibrotic matrix may induce inflammation, thereby unbalancing the expected process of vascular remodeling that guarantees the natural homeostasis of healthy capillaries. Our data suggest that this may occur by promoting abnormal secretion of new matrix and preventing controlled degradation of abnormal matrix proteins. These conditions fit within the well-reported progression of fibrotic diseases, where an increase in matrix stiffness is accompanied by inflammation and a worse prognosis of clinical outcomes, such as in breast cancer (*56*), diabetes (*57*, *58*), and cardiovascular diseases (*59*, *60*). This points to a potential target for antiangiogenic cancer therapy (*61*), where the gradual fibrosis of the premalignant microenvironment that is known to promote malignant transformation may be therapeutically intercepted before the tumor-prone region becomes cancerous.

Multiple studies have emphasized the essential role of NOTCH3 in mediating heterotypic adhesion between ECs and PCs (*21*, *45*, *62*). We postulated various regulatory mechanisms for how PCs might mediate the interaction of endothelial capillaries with the ECM. Integrin β1 has been shown to control VE-cadherin localization and blood vessel stability (*63*); however, silencing of the *TGF*β*1* gene only exacerbated the dysregulation of vascular morphology and barrier function. TGFβ1 is a key regulator of pericyte differentiation (fig. S20) (*64*). Silencing of the *TGF*β*1* gene significantly reduced the negative effects of stiff bundled collagen on vascular capillaries; however, it also had seemingly negative effects on morphology and migration patterns in soft reticular collagen, which would be undesirable from a therapeutic perspective. Silencing of *NOTCH3*, on the other hand, was postulated to block the heterotypic cell communication between ECs and PCs, thus preventing dysregulated PCs from signaling ECs from the damage on the abluminal ECM. Silencing *NOTCH3* had minimal visible effects on the soft reticular collagen vessels, which became leakier in sparse areas, as previously published (*21*). However, in stiff collagen, the silencing resulted in the normalization of vasculature and a consequential reduction in IL-8 secretion (Fig. 5, A and E). This points to a potential target in vascular therapies that may have important consequences. Blocking the mechano-paracrine feedback loop mediated by PCs may reduce the proinflammatory responses and dysregulated remodeling of the vascular matrix, which may provide an effective therapeutic target for conditions in which fibrosis and inflammation are known causes of worse prognoses.

As a limitation of this study, we acknowledge that collagen in vivo is linked to many other proteins and glycoproteins that are critical for cellular phenotypes, and the model used here does not include other important components of the ECM. Yet, cells considerably changed collagen mechanics and the structure even in the absence of the other noncollagenous molecules.

In conclusion, this study identified events and mechanisms of perivascular regulation of microvascular homeostasis in response to collagen stiffening and microarchitectural changes. Our results demonstrate that PCs may be more critical mediators of vascular remodeling and perivascular inflammation than even ECs, a conjecture that was previously unknown. PCs therefore appear to further exacerbate the effects of matrix stiffening and chronic inflammation. We suggest that these findings may shine light on vascular function, and the intersection of ECM alterations with vascular abnormalities that are common to many diseases, and may therefore have important implications for therapeutics, disease biology, and regenerative medicine.

## MATERIALS AND METHODS

### Fabrication of the microfluidic device

A CAD program was used to design a mold composed of two reservoirs connected by a central channel and a chamber. We then used a three-dimensional (3D) printer (CADworks3D μMicrofluidic printer) and resin (Master Mold resin, CADworks) to print the positive molds. Printed micromolds were cleaned in methanol in three rinses of 2 min each under agitation, subsequently were cast with PDMS (Sylgard 184, Dow-Corning), and left to cure overnight at 80°C, as previously described (*23*, *25*). Next, PDMS was removed from the resin mold, and two reservoirs were prepared using a 5-mm biopsy punch for media and 1-mm biopsy punches for the collagen loading ports. Molds were cleaned with ethanol and immediately plasma bonded to a glass coverslip. Assembled devices were autoclaved, then treated with 1% (w/v) glutaraldehyde (Sigma-Aldrich) for 15 min, rinsed three times with distilled water (DIW), and left overnight in DIW to remove any trace of glutaraldehyde. To mold cylindrical channels, sterile 160-μm-diameter acupuncture needles were immersed in a 0.1% bovine serum albumin solution for at least 30 min and then inserted into the central channel of the devices ~200 μm above the glass coverslip surface. Rat tail collagen type I (3.0 mg/ml, BD Biosciences) was prepared according to the manufacturer’s protocol.

### Collagen preparation

A stock solution of collagen type I (collagen I, rat tail Gibco, 3 mg/ml, cat. no. 10483-01) was prepared on ice with 10× phosphate-buffered saline (PBS), 1 M NaOH, and endothelial growth medium (EGM, Lonza) according to the manufacturer’s protocol. Briefly, to prepare 1 ml of a collagen solution (2.5 mg/ml), 833 μl of collagen was mixed with 100 μl of PBS, 20 μl of 1 M NaOH, and 47 μl of EGM and the working pH was 7.0 to 7.2. Thirty microliters of the final solution was immediately pipetted into the middle chamber and allowed to polymerize at 37°, 16°, 21°, or 4°C (*24*). To prevent collagen dehydration, after 1 hour, groups 37°, 21°, and 16°C had their main reservoirs filled with EGM, and then devices were returned for incubation at their respective temperatures overnight. On the following day, the cell medium was added to the reservoirs of the 4°C samples and allowed to incubate for 1 hour, then needles were carefully removed with a pair of tweezers, and the cell medium (EGM) was replaced by fresh medium. Subsequently, the devices from all groups, with their reservoirs filled with fresh EGM, were placed in the incubator at 37°C and 5% CO_2_ overnight before cell seeding.

### Scanning electron microscopy, quantification of collagen fiber thickness, and pore area

The collagen scaffold structure and morphology were analyzed via scanning electron microscopy. To that end, 8-mm collagen disks (*n* = 3) were prepared as described above, cross sectioned, fixed using a solution containing 2.5% glutaraldehyde in cacodylate buffer for 2 hours, dehydrated with graded ethanol solutions, and lyophilized overnight. Samples were subsequently sputter coated with gold/palladium and imaged using a FEI Helios Nanolab 660 DualBeam Scanning Electron Microscope at 20.0 kV or a FEI Quanta 200 SEM at 20.0 kV. For quantification of collagen fiber thickness and pore size, three ROIs (left-center-right regions of interest) from each disk were imaged. Next, ImageJ was used to measure fiber thickness and pore area.

### Atomic force microscopy

To determine the elastic modulus of the bulk collagen hydrogels, nanoindentation testing was performed with a Piuma nanoindenter (Optics11) using a 3-μm spherical tip (0.023 N/m). Hydrogel modulus was determined by fitting force-indentation curves to established models for Hertzian contact of a spherical indenter on an elastic half-space, assuming a Poisson ratio of 0.5.

The local mechanical properties of the collagen fibrils were measured by atomic force microscopy, using a Bioscope Catalyst device (Bruker Santa Barbara, CA) attached to an inverted optical microscope (IX71, Olympus, Japan) in a similar manner as described previously (*24*). Briefly, the gels were probed under PBS with a V-shaped cantilever (conically tipped, nominal radius of 10 nm, and *k* = 0.01 N m^−1^; Bruker, Santa Barbara, CA), whose spring constant was precalibrated by the thermal noise method in air. The relationship between the photodiode signal and cantilever deflection was computed from the slope of the force displacement curve obtained at a bare region of a coverslip or cell culture dish. For each gel, we used force-volume mode in which we acquired multiple force-displacement (*F*-*z*) curves (where *F* = *k* × *d*, with *d* being the deflection of the cantilever) by recording *F* and *z*, while the piezo translator was ramped downward and upward at constant speed as it moved across an area of 32 mm by 32 mm or 64 mm by 64 mm acquiring a force curve at every point of a predefined 32-by-32 array. We used 10 to 100% of the baseline to trigger force fit boundaries for acquiring the Young’s modulus values. We only included data points having an *R*^2^ value greater than 0.9 and disregarded a small fraction of force curves with lower *R*^2^ values or those that did not show a predefined trigger force of typically 1 nN. At shallow indentations (typically, a few tens of nanometers), the tip, being of scale similar to collagen fibers, will interact with individual fibers. In this case, the force-displacement (*F*-*D*) relation is linear. Thus, we can measure the effective single fiber stiffness by fitting the lower part of the *F*-*D* curves with a linear function. The slope of the fitted line is the fiber stiffness.

### Cell culture

HUVECs expressing green fluorescent protein (GFP-HUVECs) (C2519A, Lonza, Basel, Switzerland) were cultured in a supplemented (EGM-2 bullet kit, Lonza) EC growth medium (Lifeline Cell Technology, CA). hMSCs (RoosterBio, MD) were cultured in α–minimum essential medium (Gibco, Carlsbad, CA) with 10% fetal bovine serum (cat. no. 16141079, Gibco) and 1% penicillin/streptomycin (cat. no. 15140122). Cell media were changed every other day, and cells were passaged when reaching a confluency of 80 to 90%. HUVECs at passages 4 to 6 and hMSCs at passages 2 to 4 were used for all the experiments. All cells were maintained in a humidified incubator (5% CO_2_ and 37°C).

### Cell seeding

For seeding, GFP-HUVECs and hMSCs were trypsinized, counted, and mixed at a 4:1 ratio according to previous publications (*65*, *66*) in a cell density of 6 million cells/ml. Subsequently, the cell medium was removed from the reservoirs, and 25 μl of the cell suspension was added into one reservoir. The devices were flipped upside down, placed in the incubator for 5 min, seeded again, and left upside down in the incubator for another 5 min. Until the entire extension of the collagen channel had cells attached, we repeated the seeding, flipping the chip as needed. Next, the devices were placed in the incubator for 30 min under static conditions. Afterward, the devices were transferred to the two-dimensional (2D) rocker (BenchRocker) inside the incubator, as published previously (*23*, *25*).

### Cell staining and imaging

After 48 hours in culture, samples were rinsed with PBS, fixed with 10% (v/v) buffered formalin in PBS for 30 min, washed with PBS, permeabilized with 0.1% (w/v) Triton X-100 for 10 min, and blocked with 1.5% (w/v) bovine serum albumin for 1 hour under agitation. After washing with PBS, samples were incubated with one of the following primary antibodies (anti-PECAM-1, rabbit anti-human, cat. no. AB32457, Abcam, 1:100; anti-N-cadherin, rabbit anti-human, cat. no. AB18203, Abcam, 1:100; anti-laminin, rabbit anti-human, cat. no. PA1-16730, Invitrogen, 1:200; anti-NG-2, mouse anti-human, cat. no. 14-6504-82, Invitrogen, 1:200; anti-phosphorylated paxillin, rabbit anti-human, cat. no. NBP2-81063-UV, Novus Biologicals) overnight at 4°C. Samples were washed with PBS and incubated with secondary antibody (goat anti-mouse Alexa Fluor 555, cat. no. A21422, Invitrogen, 1:250; goat anti-rabbit Alexa Fluor 647, cat. no. 21244, Invitrogen, 1:250) overnight at 4°C under agitation. This was followed by rinsing in 0.1% PBS, staining of the nuclei using NucBlue [Fixed Cell ReadyProbes, 4′,6-diamidino-2-phenylindole (DAPI), cat. no. R37606, Molecular Probes], and staining of actin with ActinGreen 488 (ReadyProbes, cat. no. R37110, Molecular Probes) for 1 hour at 37°C under agitation.

Samples were imaged using a confocal microscope (Zeiss, LSM 880, Germany) with a 10× objective (numerical aperture, 0.45; Zeiss, Plan Apochromat). The depth of imaging was 100 to 400 μm, split in at least 100 *z*-stacks. *z*-Stacks were converted into TIFF files or 3D images using Zen or Imaris software (version 9.1, Bitplane, Oxford Instruments, Zurich, Switzerland). To quantify sprout density and length in the scaffolds, three images of each device were obtained. ImageJ and Imaris were used to measure the individual distances from the leading protrusions of tip cells to the wall of the parent vessel (*n* = 3 samples per condition, three ROIs per sample). The irregularity of the vessel wall and the number of migrating cells in the stiff bundled EC + PC group may give the appearance of more sprouts in this group. To ensure objectivity, we blinded the images so the operator could not see the group to which the images belonged. The operator then opened the images in Imaris and evaluated only the PECAM-1/DAPI channels to avoid confusion with the migrating cells derived from hMSCs, which were stained green. We used the slicing tool to take screenshots from three different longitudinal slices of each vessel, taken at 50, 100, and 150 μm from the vessel bottom in the *z* axis, to consider a representative quantity of sprouts per sample. We then traced a line to delimit the vessel wall and measured the individual distances from the leading protrusions of tip cells to the wall of the parent vessel for each image (fig. S25). Migrating cells were quantified using the plugin “Cell counter” from ImageJ: Only cells that had no contact with the parent vessel and did not stain for PECAM-1 were considered (*n* = 3 samples per condition, three ROIs per sample). To quantify NG2-positive cells associated with the capillaries, the images were opened in ImageJ and only cells that were in contact with the ECs were considered. For PECAM-1 and p-pax quantification, images were converted to binary, skeletonized in ImageJ, and normalized by the number of nuclei. Laminin was measured with ImageJ as a function of vessel area.

### Dextran assay for barrier function measurement

To measure the permeability of the endothelium in the microfluidic platform, fluorescent dextran (10 kDa Cascade Blue & 70 kDa Texas Red, Thermo Fisher Scientific) was introduced into perfusion media (EGM2) at a concentration of 12.5 μg/ml. Diffusion of the dextran was imaged in real time with a confocal microscope (LSM 880, Carl Zeiss) at 10× magnification. The diffusive permeability coefficient (*P*_d_) was calculated by measuring the flux of dextran into the collagen gel and fitting the resulting diffusion profiles to a dynamic mass conservation equation as described previously (*15*). Next, 70-kDa dextran was perfused through the vessel lumens, and extravasation of the dextran was measured as a function of time to quantify the diffusive permeability.

### NanoString and differential expression analysis

Single channels within the collagen (∼150,000 cells) were removed from each device, then suspended in 0.05 ml in radioimmunoprecipitation assay buffer (Thermo Fisher Scientific) at 4°C, vortexed for 10 min at 4°C, and lysed, and the RNA content was measured using a Nanodrop. Only samples with at least 100 μg of RNA content per microliter with RLT buffer (Qiagen) + 1% 2-mercaptoethanol (Sigma-Aldrich) were used. A volume of 1.5 μl of lysates was used in the NanoString hybridization reaction, and the remainder was stored at −80°C. nCounter Elements (NanoString) hybridization was performed according to the manufacturer’s instructions. The Pan Cancer Progression panel was used, and the list of gene targets is given in dataset S1.

Differential expression analysis was performed using the R package DESeq2 (v 1.24.0). The filtered count table was normalized using DESeq2’s median of ratios method of normalization. Differentially expressed genes were then identified using the negative binomial generalized linear mixed method in DESeq2 with a *P* value cutoff of 0.01 and a fold change cutoff of 2. The differential gene list was then sorted by the adjusted *P* value, and the top 50 genes were plotted on a heatmap using pheatmap (version 1.0.12). The full differential expression results were also plotted on a mean difference plot and a volcano plot using the functions Glimma MD Plot (glMDPlot) and Gimma XY Plot (glXYPlot) from the package glimma (version 1.10.1). The pathways and enrichment scores were obtained using the David Annotation tool and plotted with SRPLOT.

### siRNA transfection

hMSCs were plated into a six-well plate in antibiotic-free α–minimum essential medium (Gibco) 24 hours before siRNA transfection. Transfection reagents and siRNA for NOTCH3, TGFB1, IntegrinB1, and ON-Target plus cyclophilin B control pool (ON-TARGET plus siRNA kits, Horizon Discovery, cat. nos. L-011093-00-0005, L-012562-00-0005, L-004506-00-0005, and D-001820-10-05) were diluted in Opti-MEM and added to the wells of 80% confluent cells, according to the manufacturer’s instructions (*21*). Each gene was silenced in different well plates to avoid any cross contamination during the procedures. Next, 2 μl of transfection reagent was used per well, and siRNAs were added for a final concentration of 40 nM. After 24 hours, cells were passaged and used in downstream experiments. Cells were lysed for Western blot at 48 hours after transfection to quantify knockdown. This time point coincided with the end point of the single-channel experiments. Three independent experiments were performed, and the graphics represent quantification of these independent samples.

### Engineering bone marrow, seeding on the vascularized chip, and flow cytometry

Bone marrow organoids were fabricated by seeding 3 × 10^3^ mesenchymal stem cells (hMSCs, Lonza, PT-2501) and 6 × 10^3^ human bone marrow CD34+ cells (STEMCELL Technologies, 70002.3) into a collagen-based hydrogel according to the protocol described previously (*67*). These samples were cultured for 7 days in StemSpan SFEM II (STEMCELL Technologies, 09655) supplemented with a cytokine cocktail that supports hematopoietic stem cell growth and differentiation, the basis of which is described in (*67*). After 7 days of culture under standard conditions, cells were harvested from the 3D matrix using collagenase type I (Gibco, 17018-029) and NSK fermented soybean extract (Japan Bio Science Laboratory Co., Ltd., NSK-SD). A total of 6 × 10^3^ cells were reseeded into an identical hydrogel.

Next, microfluidic devices for the single channels had the top of the collagen chamber removed with a 6-mm biopsy punch, and then hollow channels were fabricated with acupuncture needles and soft reticular or stiff bundled collagen as described above. GFP-HUVECs and hMSCs were seeded, and after 24 hours, a bone marrow–loaded hydrogel was overlaid on the vascularized chip inlet well and solidified at 37°C (fig. S20). The chip was cultured for 72 hours on a 2D rocker under standard conditions in the supplemented StemSpan SFEM II media. Cell media were changed every day.

Before flow cytometry, the hydrogels were digested with collagenase type I (Gibco, 17018-029) and NSK fermented soybean extract (Japan Bio Science Laboratory Co., Ltd., NSK-SD). The cells were stained with Live/Dead Green Fixable Dead Cell Stain (Invitrogen, L34970), PE Mouse Anti-Human CD16 (3G8, BD Biosciences), BV421 Mouse Anti-Human CD33 (WM53, BD Biosciences), APC Mouse Anti-Human CD34 (581, BD Biosciences), PerCP-Cy5.5 Mouse Anti-Human CD45 (HI30, BD Biosciences), APC/Cyanine7 anti-human CD90 (5E10, BioLegend), PE-Cy5 Mouse Anti-Human CD235a (HIR2, BD Biosciences), APC-R700 Mouse Anti-Human CD117 (YB5.B8, BD Biosciences), and BUV805 Mouse Anti-Human CD14 (MφP9, BD Biosciences). Stained cells were washed and resuspended with CountBright Absolute Counting Beads (Invitrogen, C36950) and Flow Cytometry Staining Buffer (Invitrogen eBioscience, 00422226). Data were collected using a Cytek Aurora 5-laser spectral cytometer and SpectroFlo software (Cytek). The unmixed data were gated and analyzed with FlowJo version 10.9.0. The cells of interest were isolated by gating out debris, doublets, and dead cells. Monocytes were identified as CD45^High^ CD33^High^ CD34^−^ CD117^−^ CD16^−^ CD14^+^. Neutrophils were identified as CD45^High^ CD14^−^ CD34^−^ CD33^+/−^ CD16^+^. Mesenchymal stem cells were identified as CD45^Low^ CD235a^−^ CD34^−^ CD90^+^ cells. The distinction between CD33^High^ and CD33^Low^ cells was made downstream of CD45^High^.

### Enzyme-linked immunosorbent assay (ELISA)

The IL-8 and TGFβ1 production was measured from the supernatants of three biological replicate chips from soft reticular and stiff bundled groups after 48 hours of incubation. The concentration of both cytokines was determined by a RayBio Human IL-8 ELISA Kit (RayBiotech, Peachtree Corners, GA) and RayBio Human TGFβ1 ELISA Kit (RayBiotech, Peachtree Corners, GA) following the manufacturer’s instructions. The results were compared to a colorimetric standard curve (4000 to 16.38 pg/ml for TGFβ1 and 600 to 0.8 pg/ml) after reading the microplates in a microplate reader (SPARK 20M, TECAN, Seestrasse, Männedorf, Switzerland).

### Statistical analysis

All experiments were done at least in triplicate. Data are presented as means ± SD, and statistical analyses were performed using GraphPad Prism (version 9, GraphPad Software, LLC) using one-way or two-way analysis of variance (ANOVA) and Tukey’s post hoc tests (α = 0.05). For pairwise comparisons, Student’s *t* test was used. Statistically significant differences were determined as *P < 0.05, **P < 0.01, ***P < 0.001, and ****P < 0.0001.
