# Supplementary material for: Perivascular cells function as key mediators of mechanical and structural changes in vascular capillaries
Source: Sci Adv. 2025 Jan 10;11(2):eadp3789. doi: 10.1126/sciadv.adp3789 (PMC11721577; doi:10.1126/sciadv.adp3789)
Supplement: Supplementary file 1 — Figs. S1 to S25 Legends for movies S1 to S10 Legend for dataset S1 [file sciadv.adp3789_sm.pdf]

Science Advances
AAAS

Supplementary Materials for

Perivascular cells function as key mediators of mechanical and structural changes in vascular capillaries

Cristiane M. Franca *et al.*

Corresponding author: Luiz E. Bertassoni, bertasso@ohsu.edu

Sci. Adv. **11**, eadp3789 (2025)
DOI: 10.1126/sciadv.adp3789

The PDF file includes:

Figs. S1 to S25
Legends for movies S1 to S10
Legend for dataset S1

Other Supplementary Material for this manuscript includes the following:

Movies S1 to S10
Dataset S1

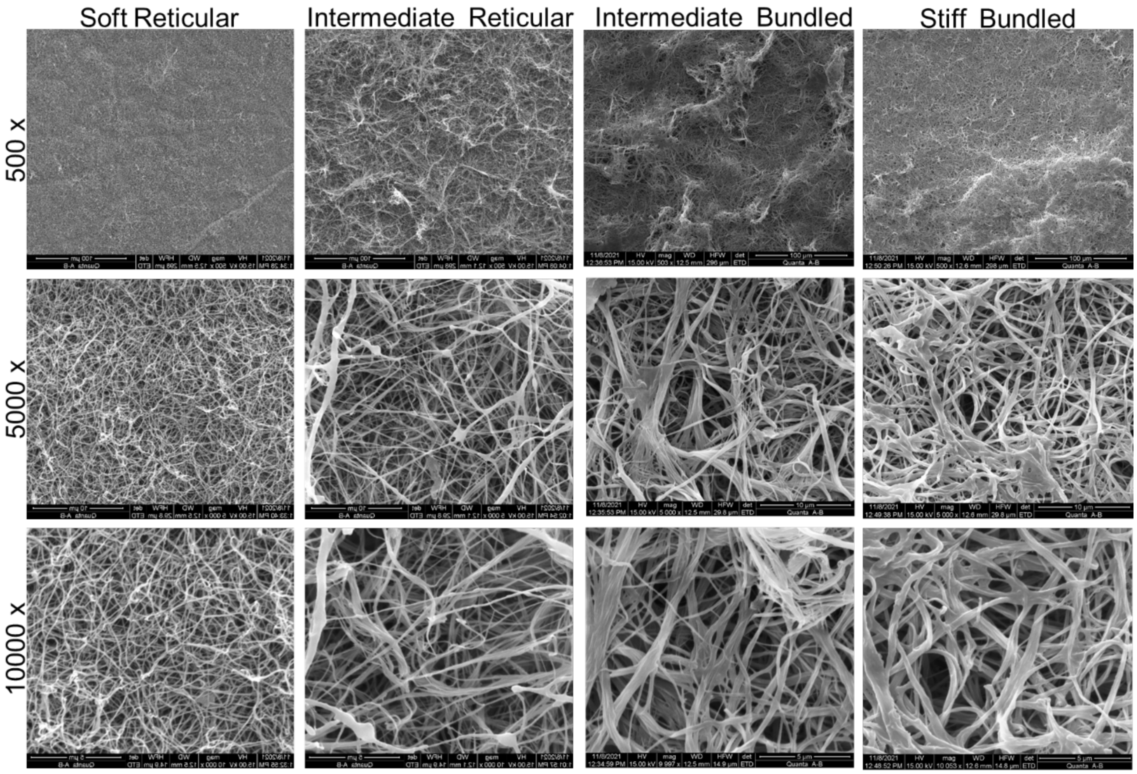

Fig. S1. SEM of collagen microarchitectures as a function of fibrillogenesis temperature. From left to right – soft reticular (37ºC), intermediate reticular (21ºC), intermediate bundled (16ºC) and stiff bundled (4ºC). Scanning electron microscopy (SEM) images show that polymerization at higher temperatures results in a soft reticular collagen with delicate fibers and homogeneous pore distribution. As polymerization temperature is decreased, fiber thickness and pore sizes increase in a controllable manner, progressively bundling collagen fibers without changing collagen density and the presence of cell ligands.

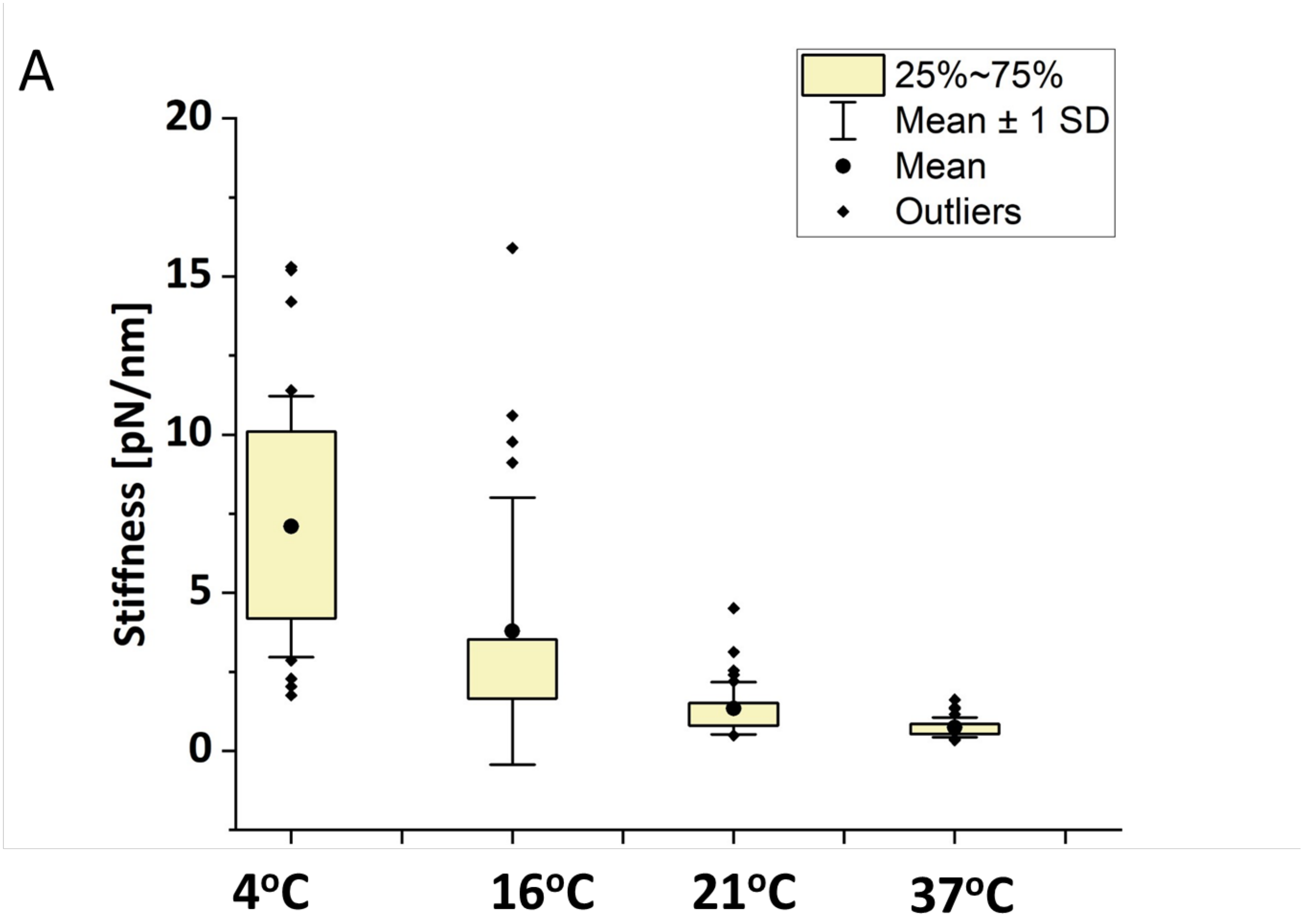

Fig. S2 According to AFM measurements, collagen polymerized at 37ºC presents single fiber stiffness at an average range of 0.73±0.3 pN/nm with the least variability. Decreasing assembling temperatures correlate with incremental increases in single fiber stiffness and greater variability among the fibers.

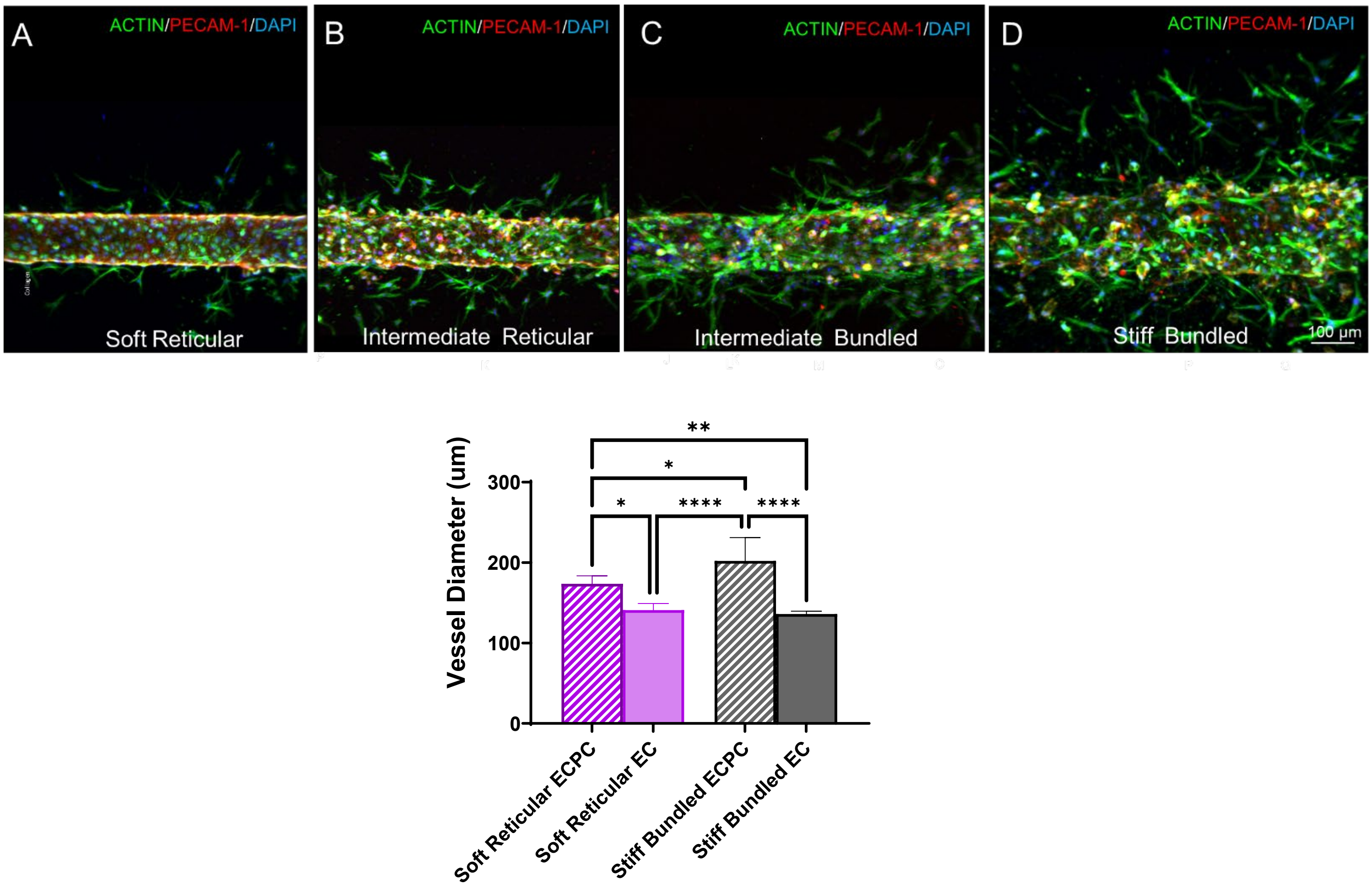

Fig. S3. Progressive changes in vascular morphology as a function of collagen stiffness and microarchitecture. (A-D) Images depicting vasculature engineered with endothelial and perivascular cells showing a progressive alteration in vascular morphology and cell migration in relation to varying collagen stiffness and microarchitecture. (B) The association of perivascular cells (PC) with stiff bundled collagen results in an increased vessel diameter (n=8). In contrast, vasculature constructed solely with endothelial cells (EC) exhibited consistent diameter regardless of the collagen's composition (n=8). One-way ANOVA post hoc Tukey, * $p<0.05$, **$p<0.01$, ****$p<0.0001$.

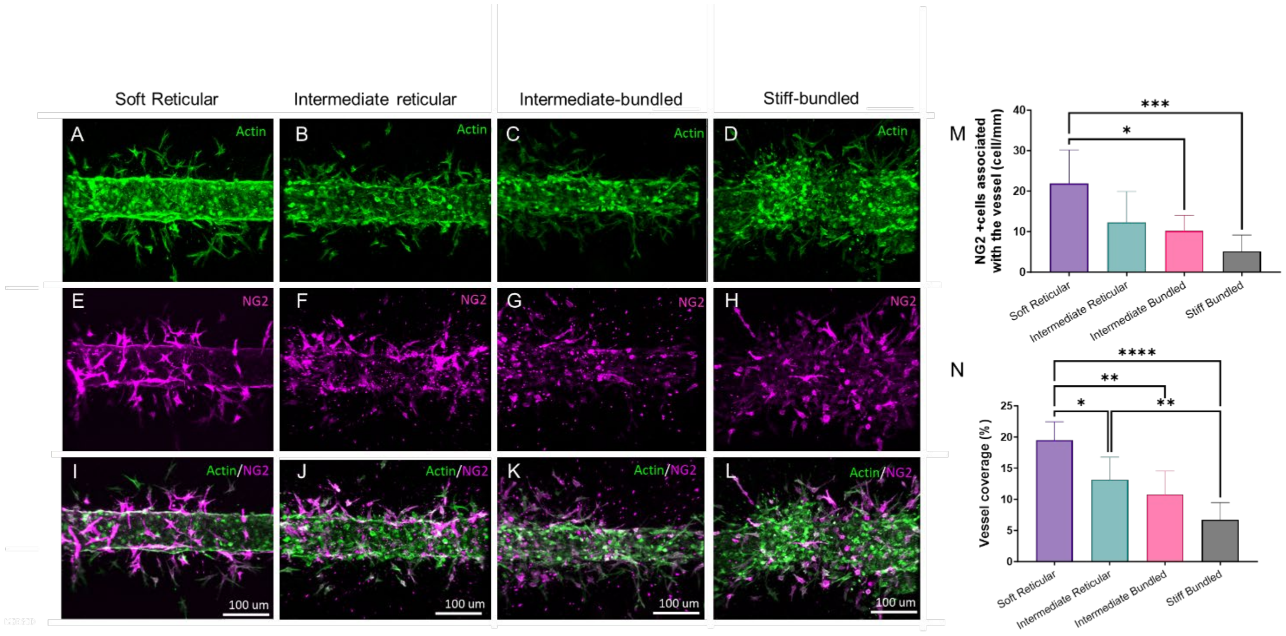

Fig. S4. **Pericyte coverage as a function of collagen stiffness and microarchitecture**. Representative images depicting a progressive reduction in NG2 (pericyte marker) across varying collagen stiffness levels. (A, E, I) Soft reticular collagen resulted in NG2 positive (NG2+) cells associated with the endothelial cells (D, H, L). On the other hand, in stiff bundled collagen, NG2+ cells migrated further from the capillaries, which are not characterized as pericytes due to their lack of contact with endothelial cells. Intermediate states (B, C, F, G, J, K) showed a progressive increase and migration of NG2+ cells away from the vasculature. (M, N) Quantitative analysis revealed a marked increase in pericyte differentiation within soft reticular collagen. Specifically, only NG2+ cells proximal to the vascular wall were identified as functional pericytes providing vessel coverage, with capillaries engineered within softer collagen exhibiting the highest pericyte coverage. All experiments were done at least in triplicate. One-way ANOVA post hoc Tukey, * $p<0.05$, ** $p<0.01$, *** $p<0.001$, **** $p<0.0001$.

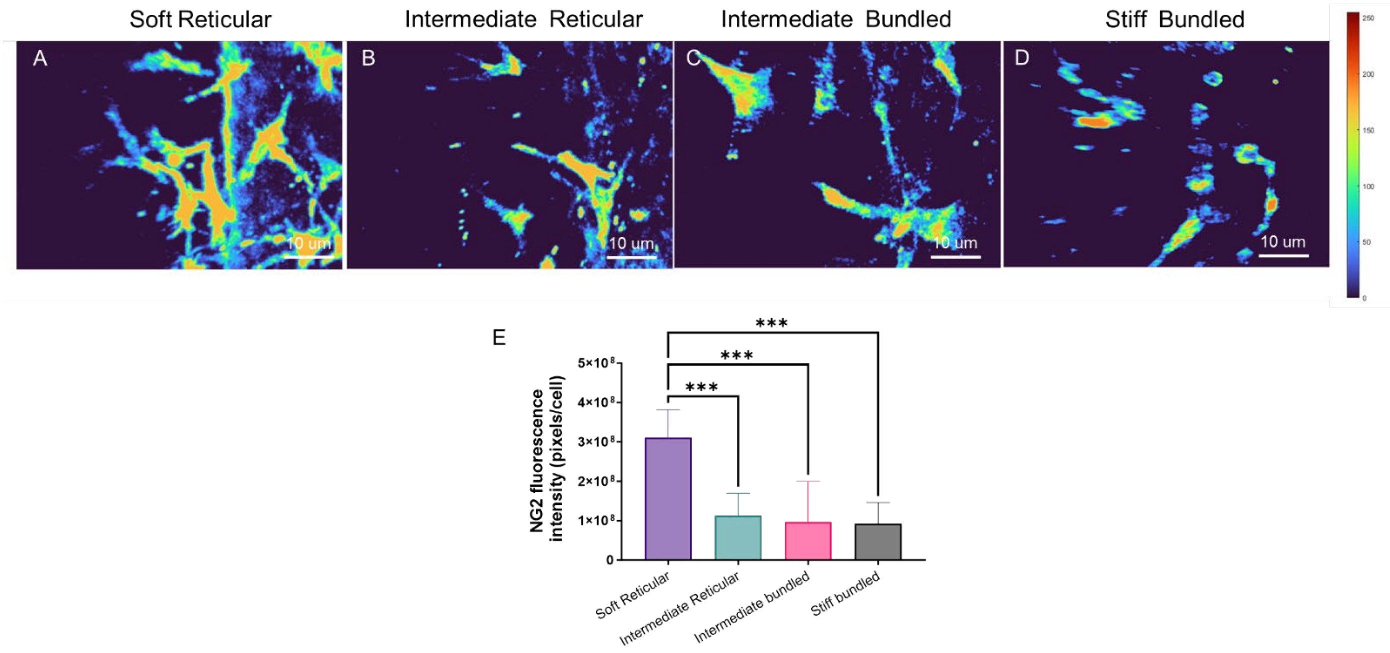

Fig. S5. **Intensity of NG2 expression**. 3D images of the perivascular cells near the capillary wall were first converted into maximum intensity projection images using Zen Blue software. The images were then imported into Imaris, where a heat map of the fluorescence intensity of NG2 labelling was generated. Each pixel had a specific color and intensity. Only the color channel corresponding to the Alexa Fluor 647 (NG2 fluorophore) was used in this quantification. The heat map represents a scale of 0-240 in pixel intensity. (A) In addition to a higher number of perivascular cells expressing NG2, vascular capillaries engineered in soft reticular collagen also demonstrated a higher intensity of NG2 expression per cell than (B,C) intermediate states or (D) stiff bundled collagen. (E) Quantification was performed using Imaris to evaluate pixel intensity per perivascular cells that were associated with the capillaries. All experiments were done at least in triplicate. One-way ANOVA post hoc Tukey, ***$p<0.001$.

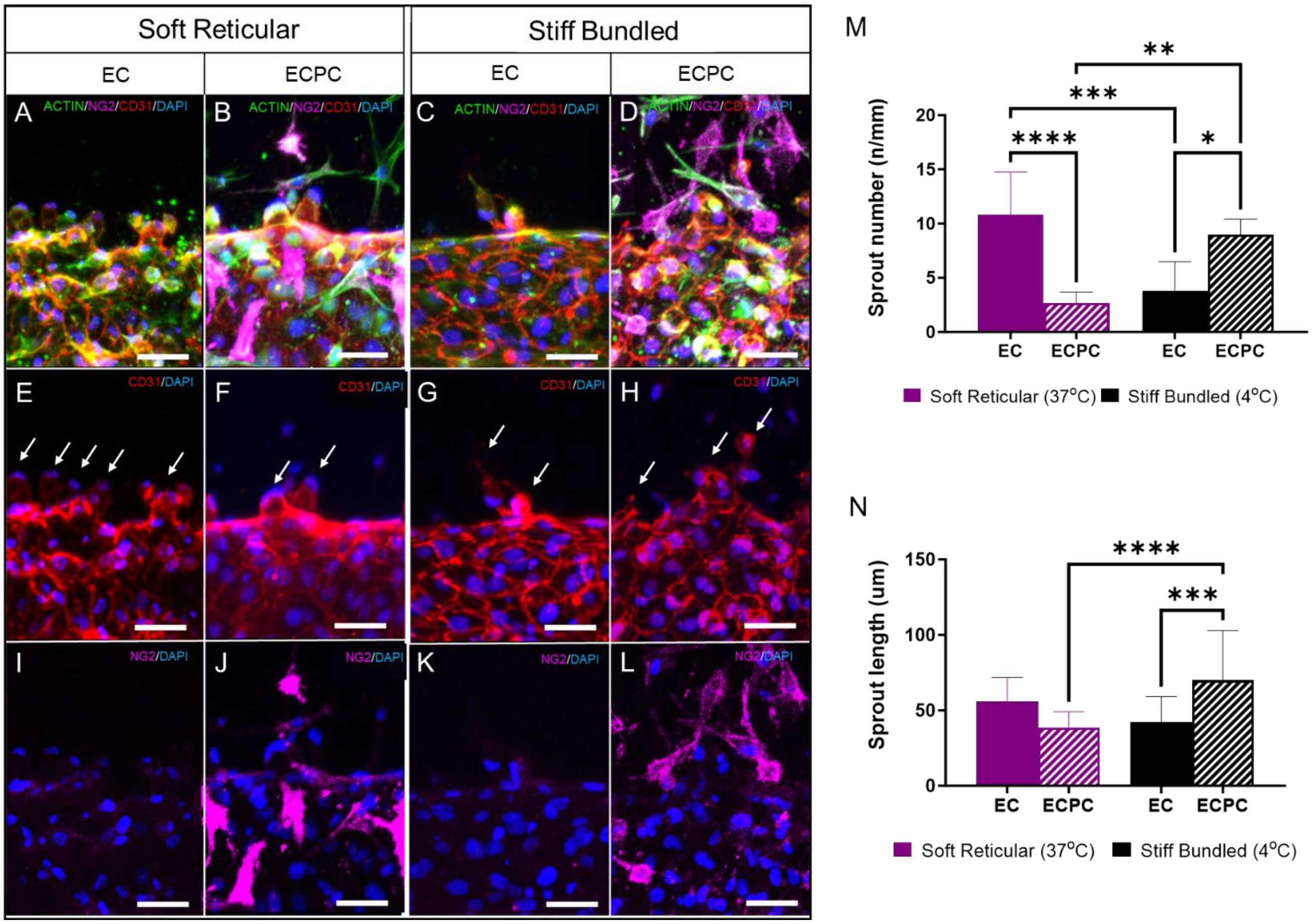

Fig. S6. **Sprouts according to the presence of perivascular cells and collagen stiffness**. Comparative analysis of sprouting angiogenesis (arrows) reveals distinct outcomes in collagen matrices based on the presence or absence of perivascular cells (PC). (A, E, I, C, G, K, M, N) The absence of PC exhibited a higher sprout count in soft (healthy) collagen matrices compared to stiff (fibrotic) ones, while sprout length remained consistent across all groups. In contrast, the presence of PC (B, F, J, D, H, L, M, N) correlated with increased sprout number and length specifically within the stiff group, indicating differential responses in the presence of perivascular cells.

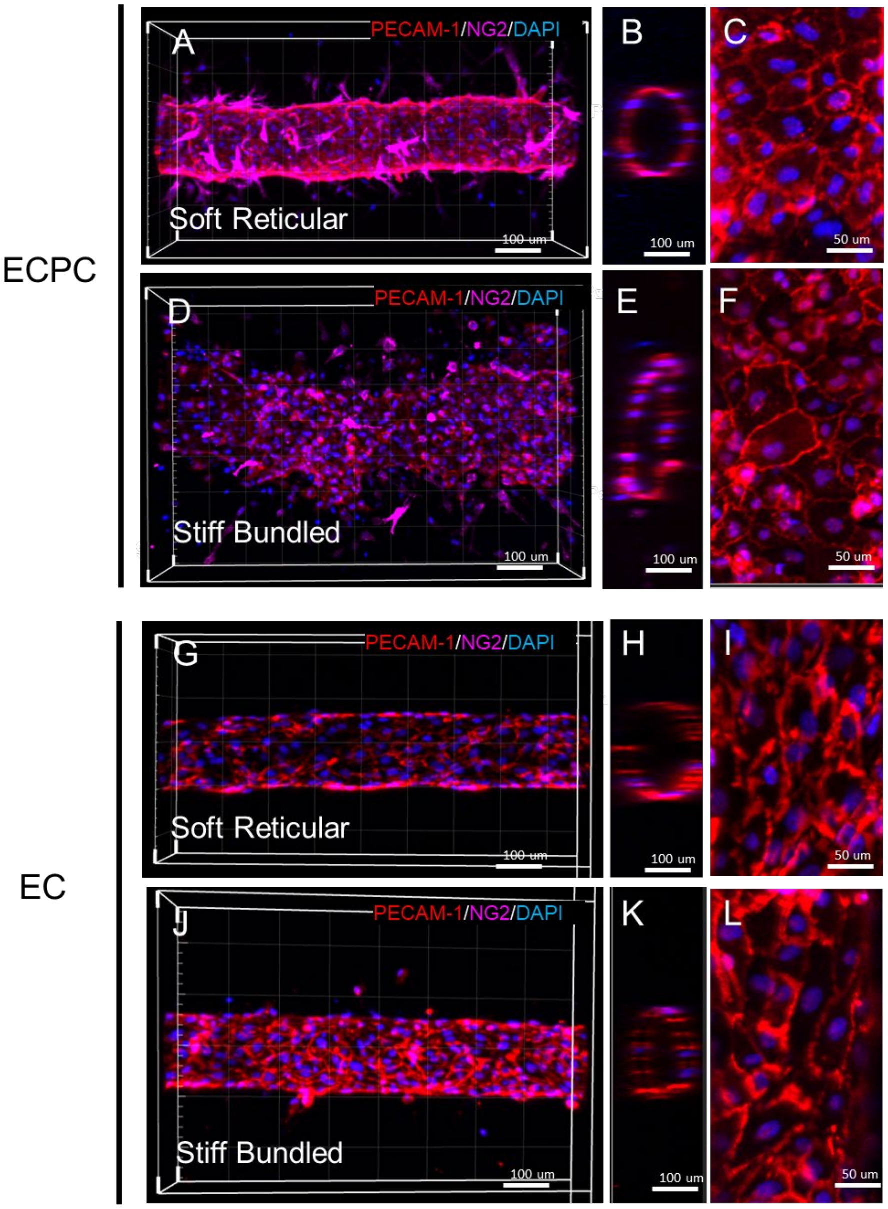

Fig. S7. PECAM-1 expression in capillaries engineered within soft reticular or fibrillar bundled collagen as a function of the presence of perivascular cells. (A-C) Endothelial cells size was more homogeneous in the soft reticular collagen (healthy) in the presence of perivascular cells. (D-F) Stiff bundled collagen (more fibrotic) and perivascular cells led to irregular cell morphology with variable size and PECAM-1 expression. In contrast, when the vasculature was engineered with endothelial cells alone, the differences between cells in the two collagen microenvironments were mitigated (G-L)

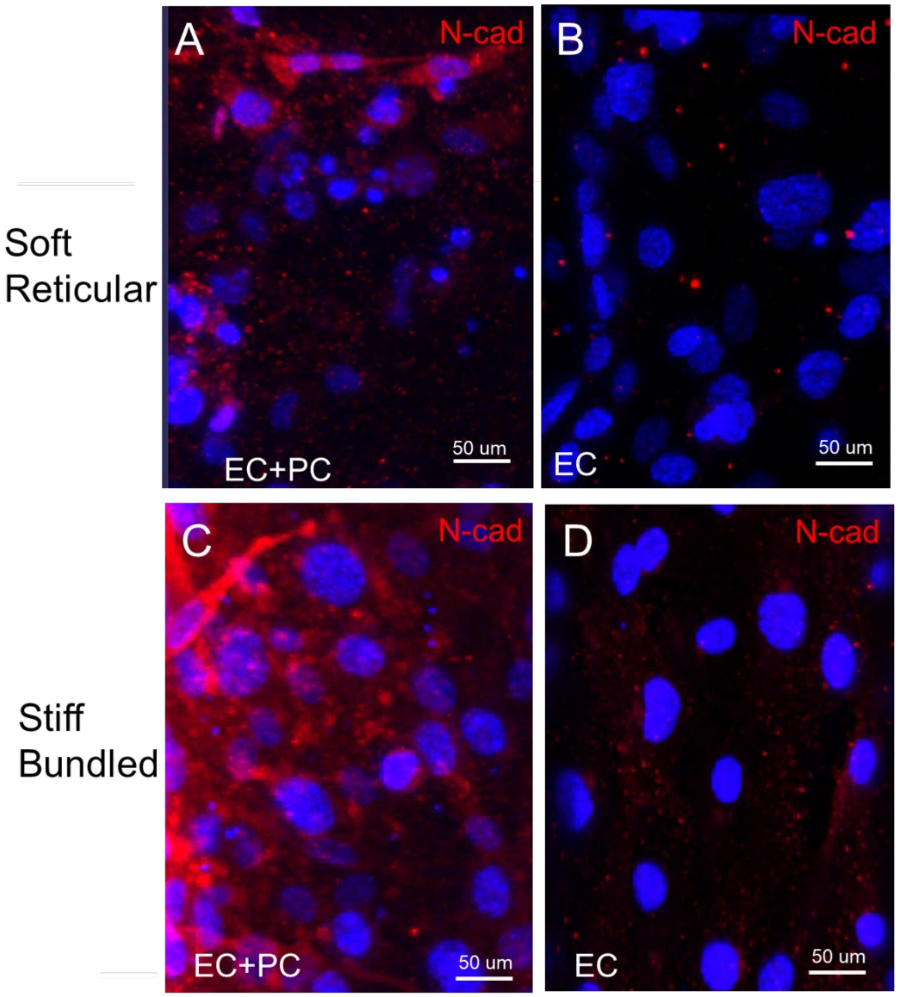

Fig. S8. Immunofluorescence analysis depicting N-cadherin expression patterns as a function of perivascular cells and collagen status. N-cadherin expression was prominently observed in the stiff bundled collagen group with perivascular cells (PC), while in the soft reticular group, it was predominantly confined to perivascular cells. Notably, in the absence of perivascular cells, endothelial cells (EC) in the stiff bundled group displayed limited N-cadherin expression.

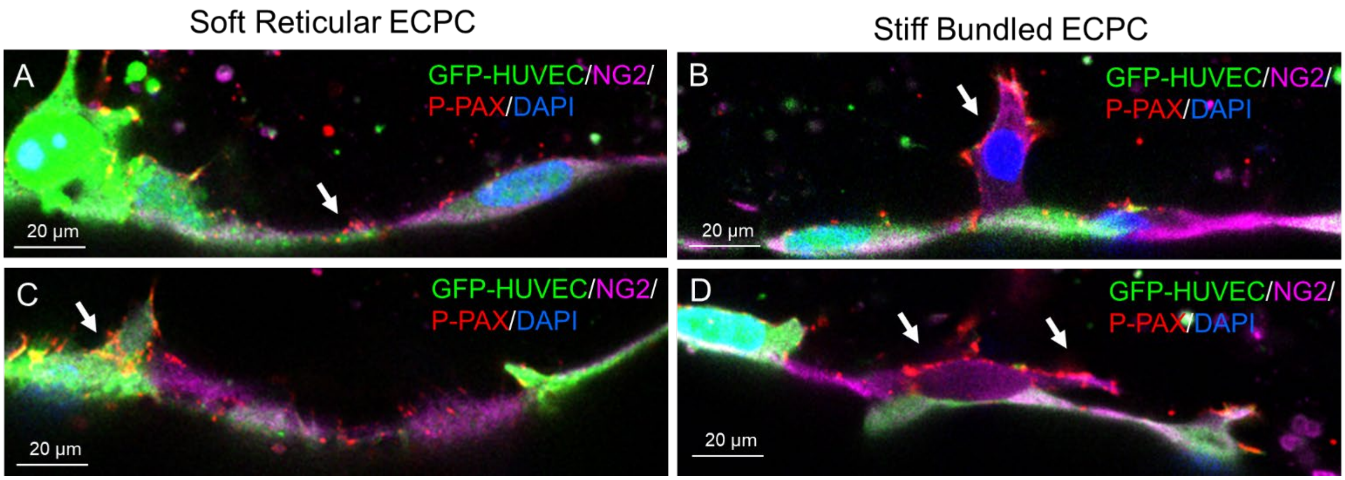

Figure S9: Differential localization of phosphorylated paxillin in pericytes according to the collagen properties. (A, C) In the vasculature engineered in soft reticular collagen, p-pax is observed as small clusters evenly distributed in both endothelial and perivascular cells. (B, D) In the vasculature engineered in the stiff bundled collagen, p-pax tended to be concentrated in the perivascular cells.

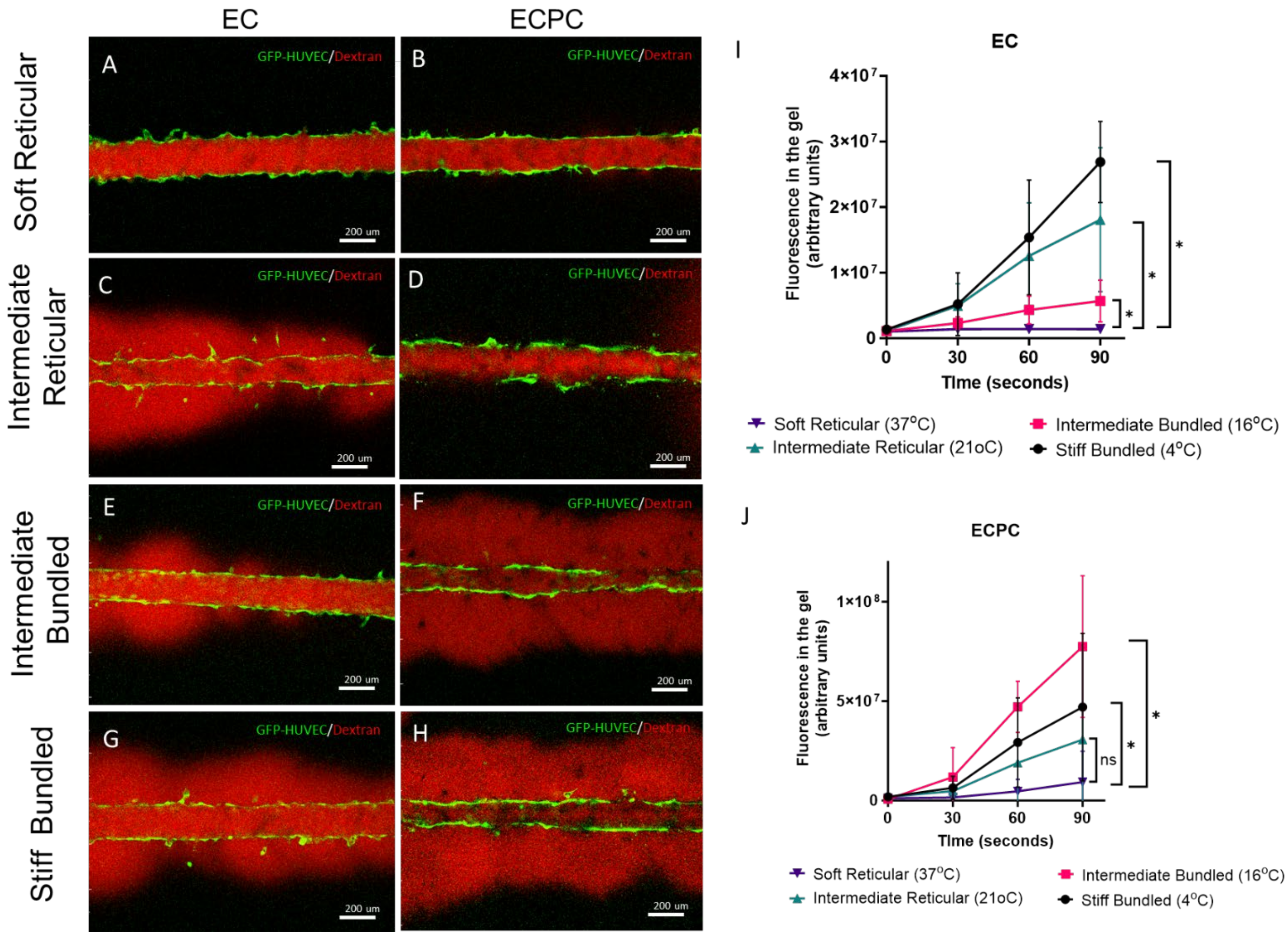

Fig. S10. Barrier function associated with perivascular cells in different collagen stiffness and architecture. Representative images demonstrating dextran assay results after perfusion. Preservation of barrier function was evident in soft reticular collagen, irrespective of perivascular cell presence (A, B). In contrast, the absence of perivascular cells led to the loss of barrier function in all other groups without PC (C, E, G) and in the intermediate and stiff bundled groups with PC (F, H). Notably, perivascular cells exhibited sustained barrier function, even with a minor stiffness increment (D). However, in collagen configurations with increased stiffness and bundling, the presence of perivascular cells proved insufficient to maintain barrier function (I, J). One-way ANOVA, $p<0.05$.

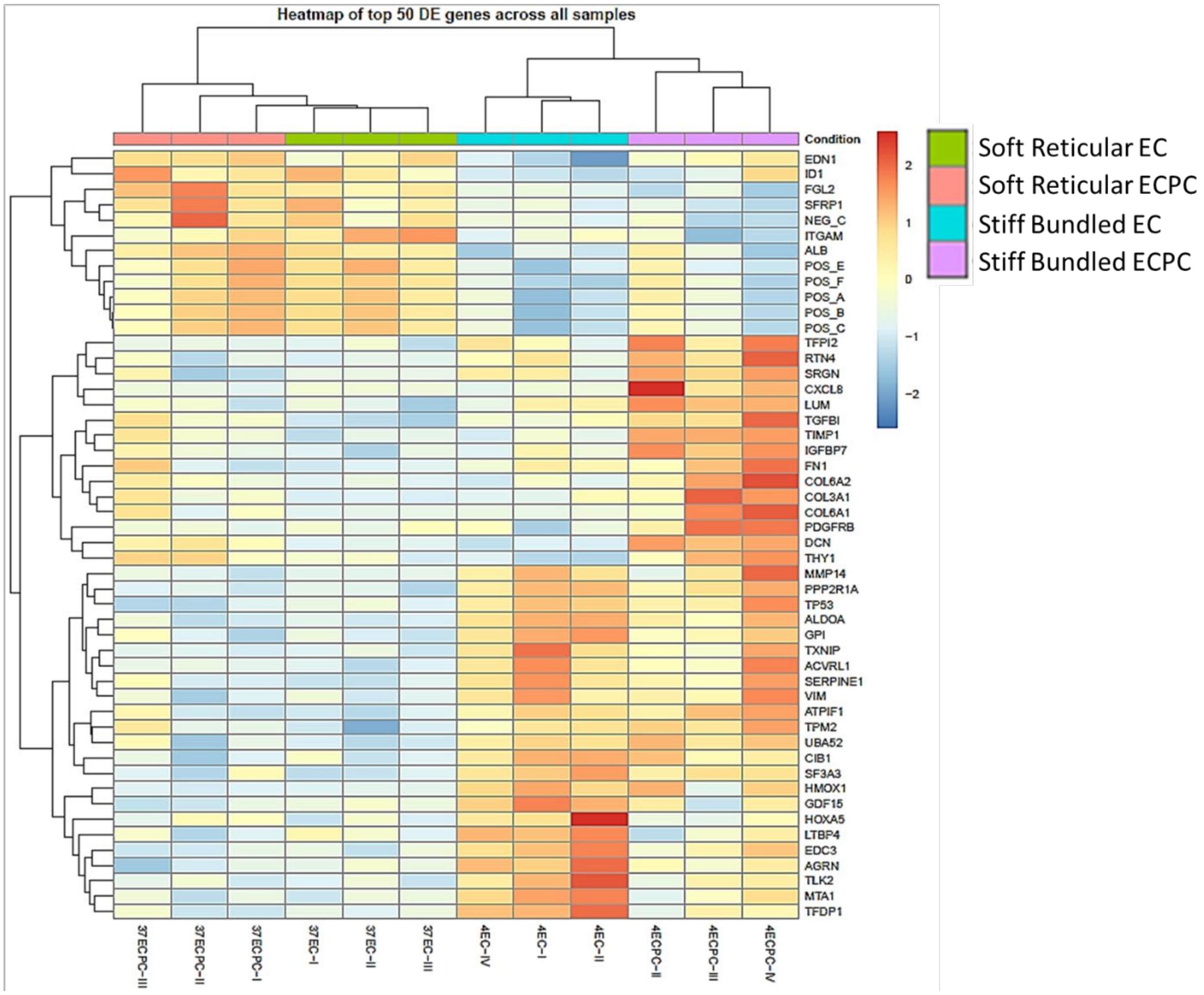

Fig. S11. **Heat map highlighting the 50 most differentially expressed genes in stiff (more fibrotic) and soft (healthy) collagen vasculature with and without perivascular cells.** Analysis of gene expression profiles demonstrates distinct patterns in different collagen environments. Stiff collagen with perivascular cells exhibits upregulation of genes including *CXCL8, FN1, DCN, COL3A1, COL6A1, MMP14*, and *PDGFRb5*. Conversely, in the absence of perivascular cells, stiff collagen shows increased expression of *AGRN, MTA, TFDP*, and *HOXA5*. Soft collagen, on the other hand, displays a smaller subset of differentially expressed genes, including *ID1, FGL2,* and *ITGAM*.

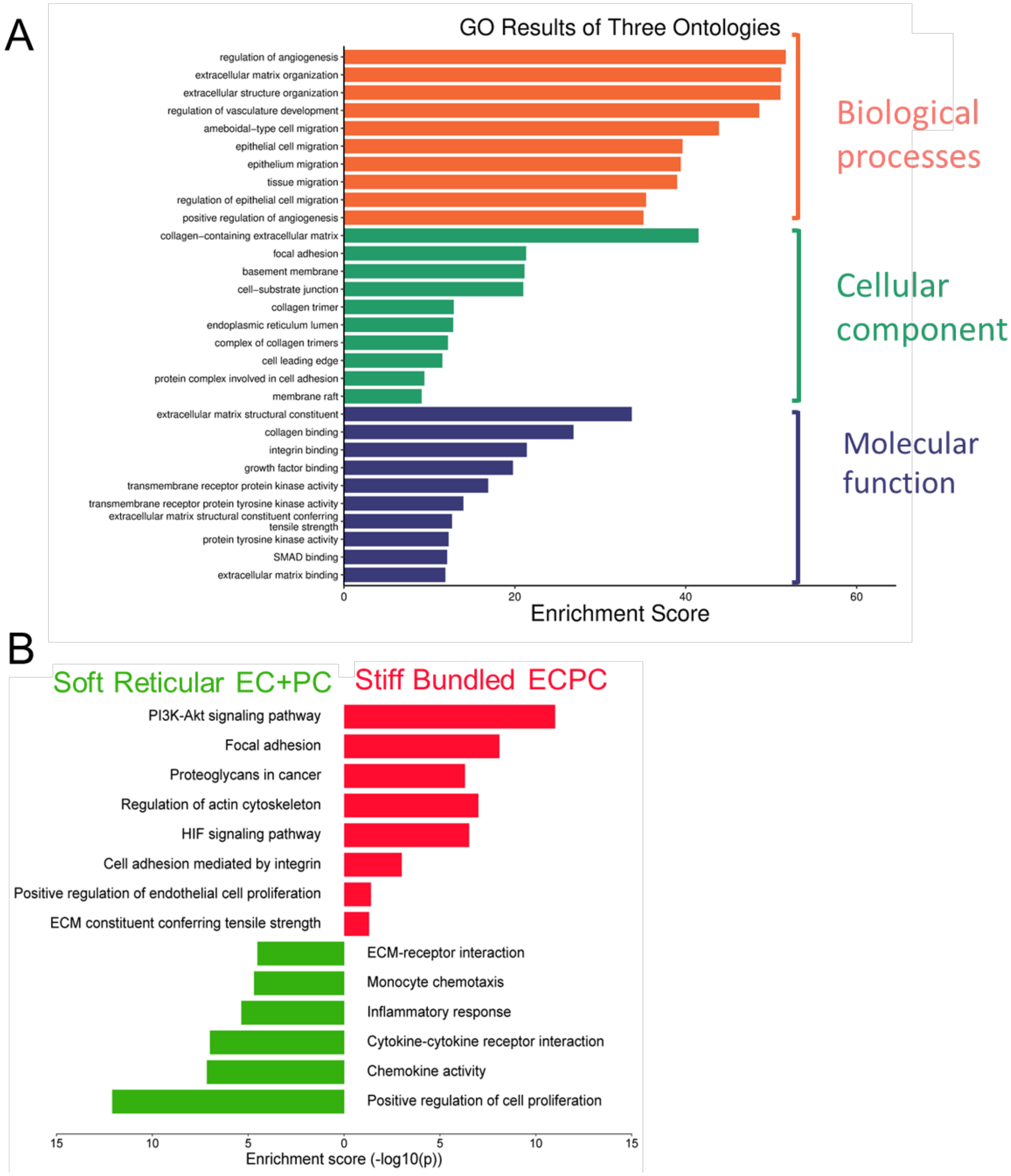

Fig. S12. **Enrichment score of the underscored pathways comparing soft reticular and stiff bundled collagen with perivascular cells.** (A, B) Most of the pathways were related to extracellular matrix interactions and different types of cell migration, which correlate with the results observed in the immunofluorescence images.

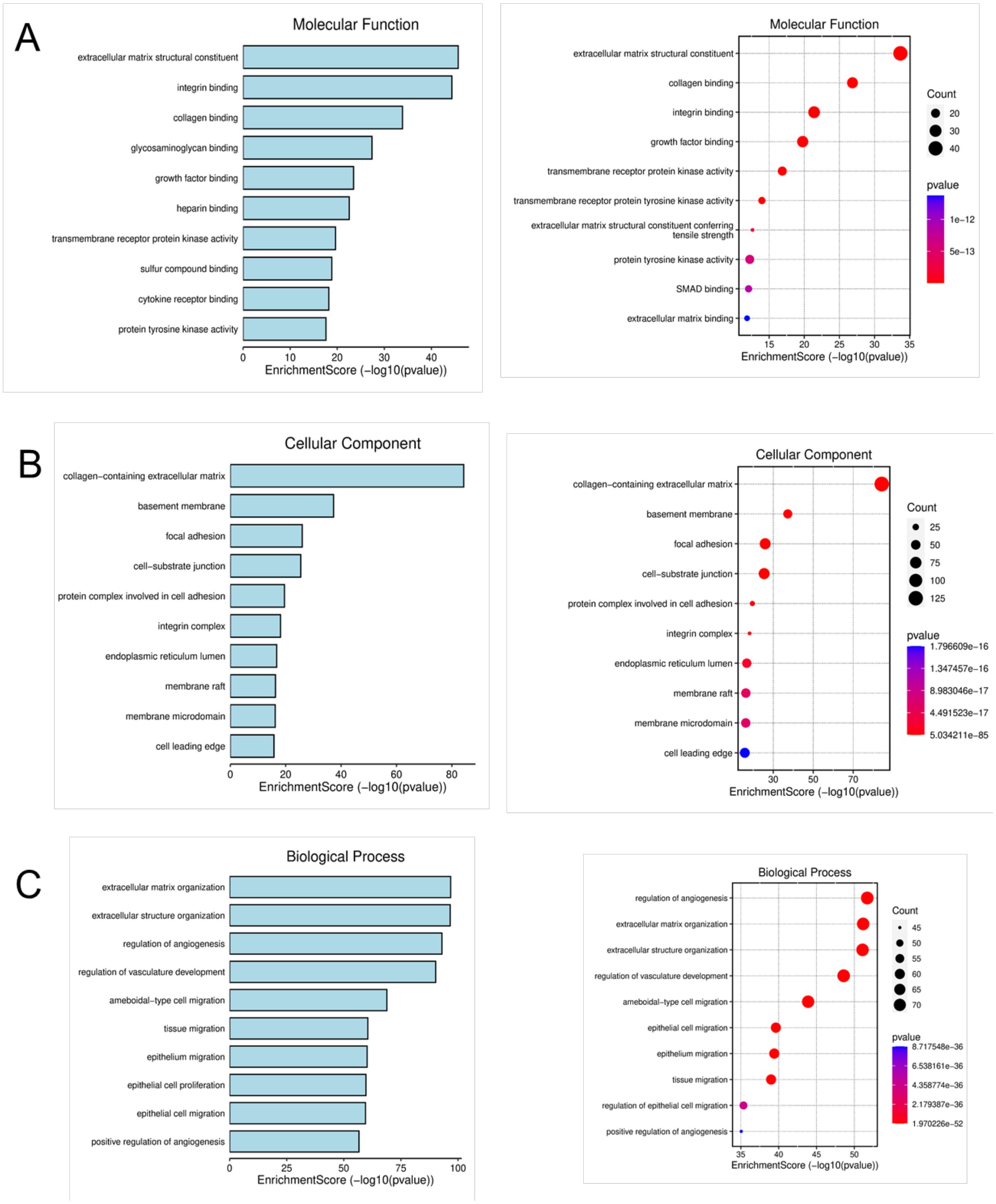

Fig. S13. Enrichment scores for capillaries engineered with perivascular cells within either soft reticular or stiff bundled collagen. (A) molecular function, (B) cellular components and (C) biological processes show pathways related to cell-extracellular matrix binding, cell migration and angiogenesis are the most activated in the vasculature engineered in the stiff bundled collagen.

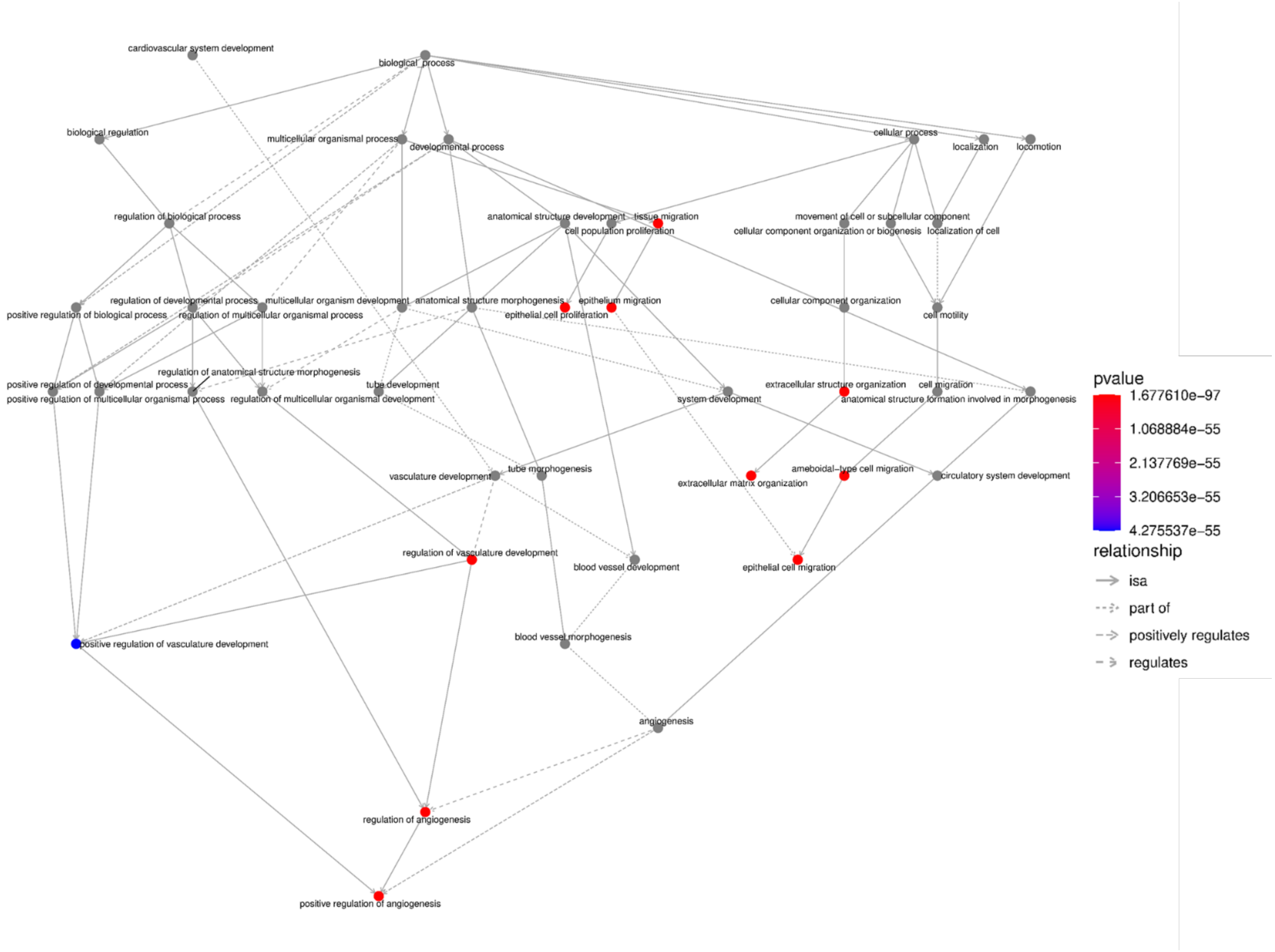

Fig. S14. **Pathways activated in capillaries with perivascular cells within stiff bundled collagen**. Data from Nanostring analysis showed that the mostly activated. pathways were related with positive regulation of angiogenesis, ameboidal-type cell migration, and extracellular matrix organization.

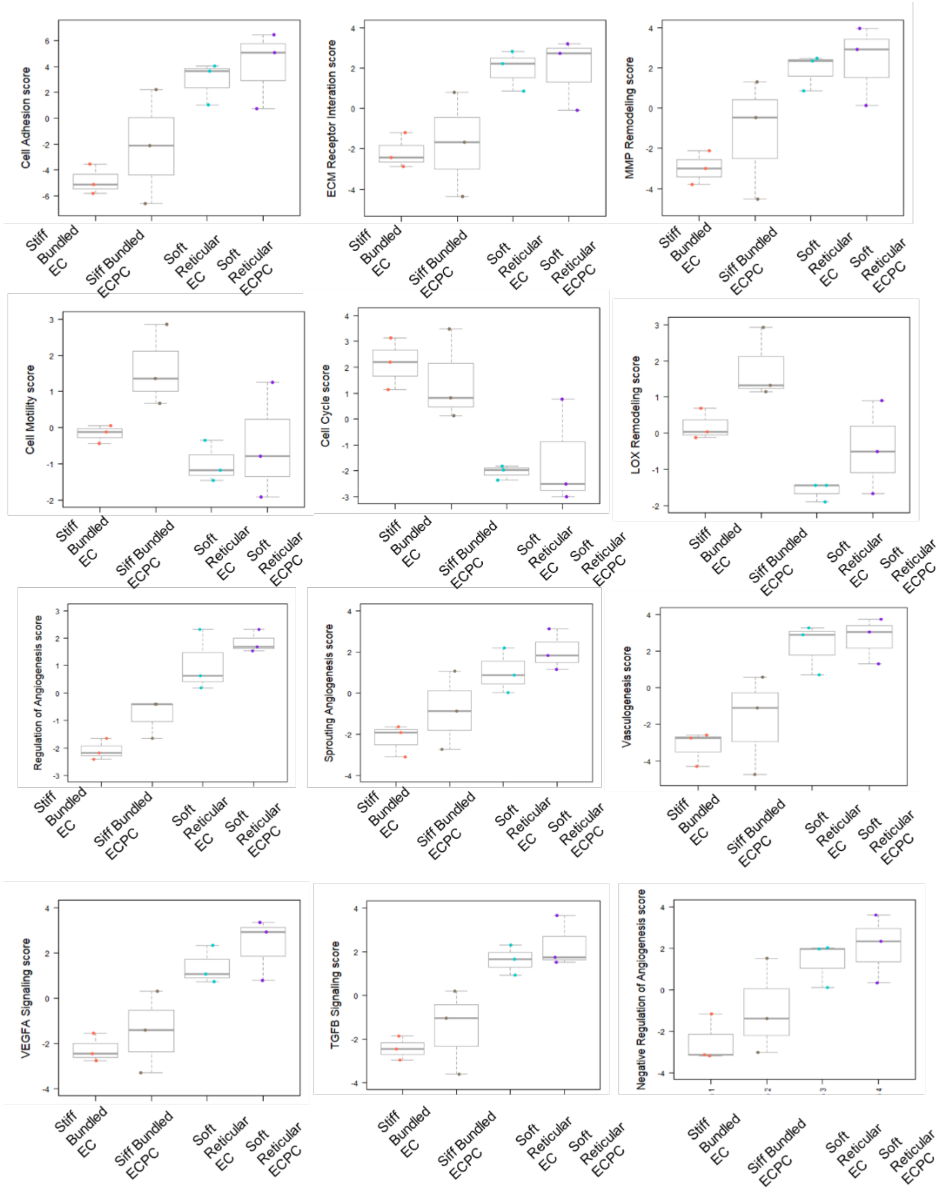

Fig. S15. **Comparison of pathways scores among the presence of perivascular cells (PC), more fibrotic (stiff bundled) and soft reticular (healthy) collagen**. Rosalind (Nanostring ^{TM}) output of data highlighting the mostly differentially expressed enrichment scores.

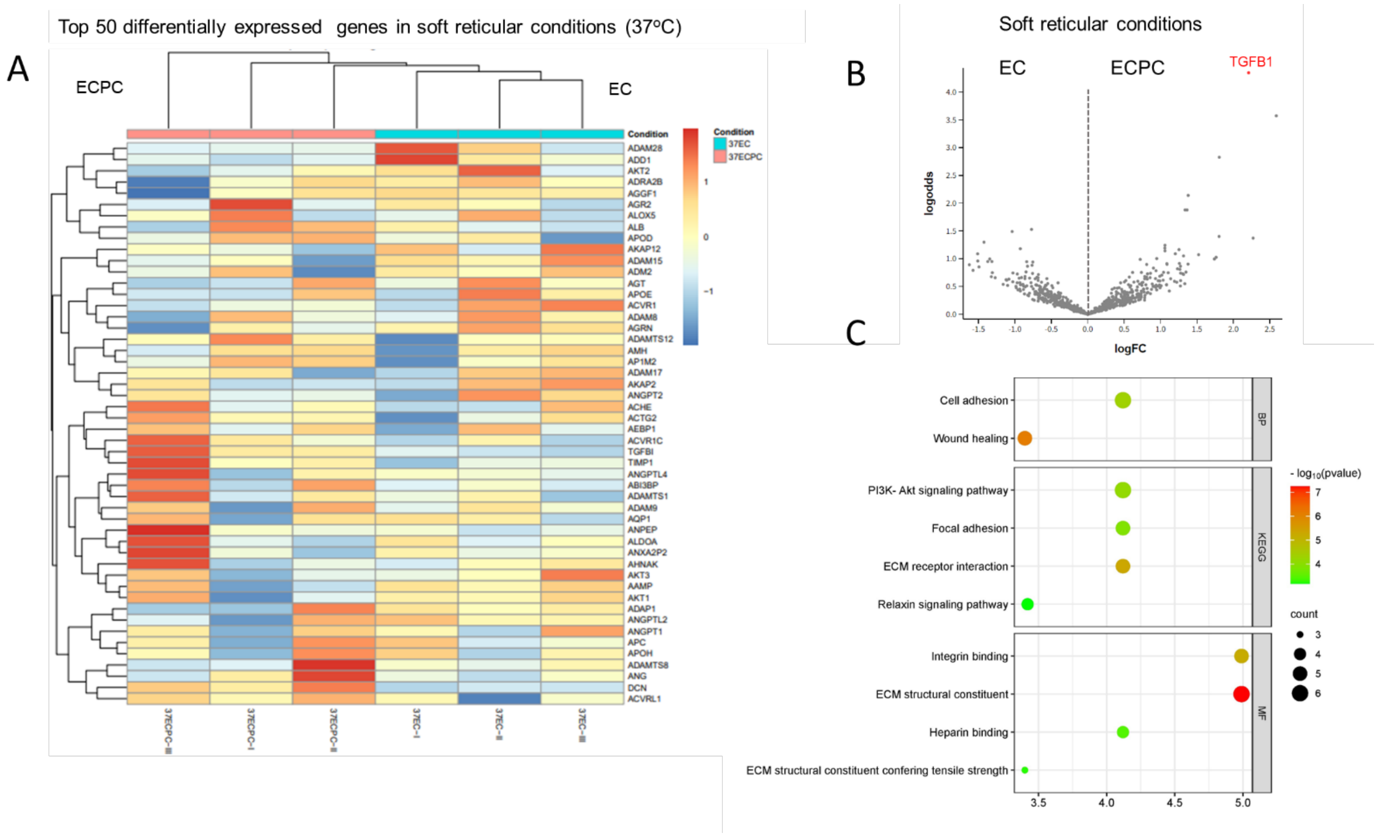

Fig. S16. **Comparison of 50 most differentially expressed (DE) genes in soft reticular (healthy) collagen vasculature with and without perivascular cells.** (A) heat map and (B) volcano plot show that the mostly differentially expressed gene was transforming growth factor beta 1 (*TGFβ1*). (C) The pathway analysis showed an increase in cell adhesion, focal adhesion, ECM receptor interaction, integrin binding and ECM structural component in the presence of perivascular cells.

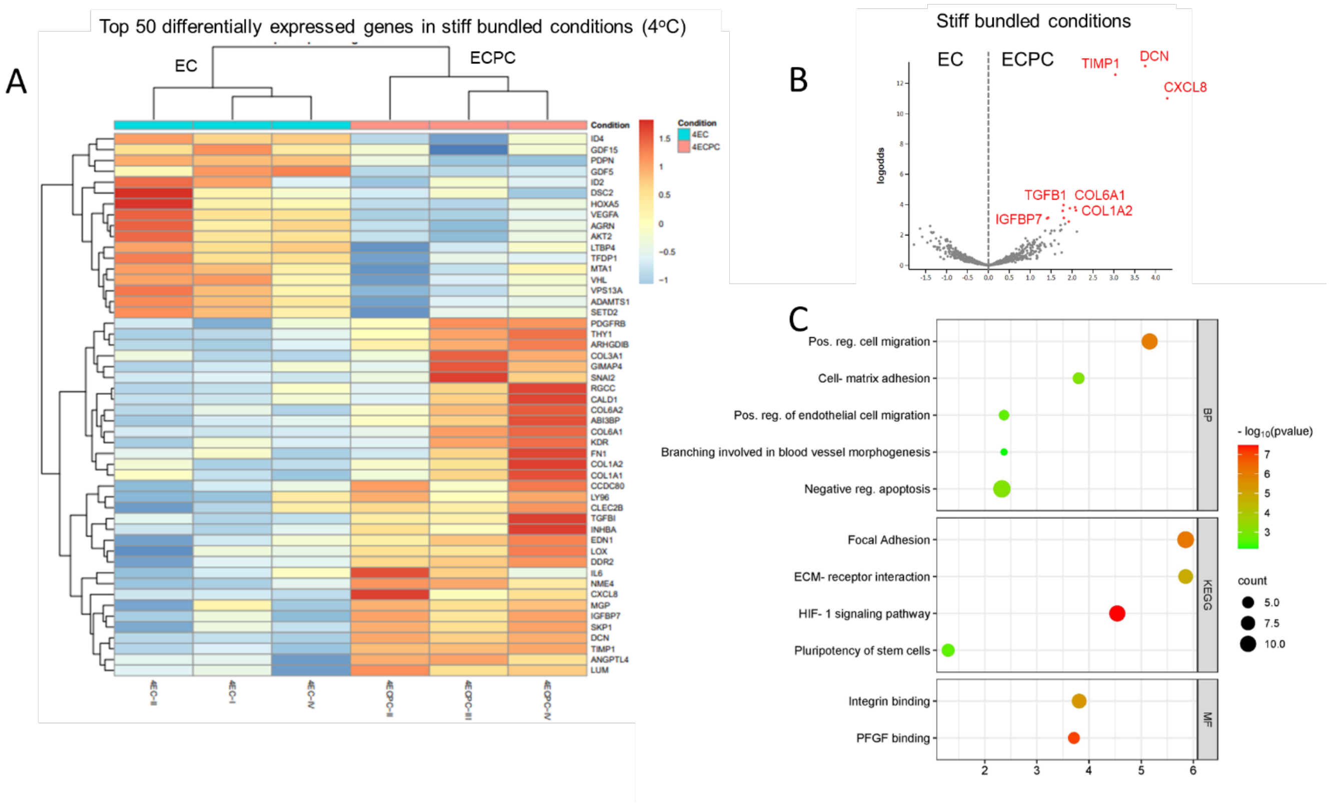

Fig. S17. Comparison of 50 most differentially expressed genes in stiff bundled (more fibrotic) collagen vasculature with and without perivascular cells. (A) Heat map and (B) volcano plot highlight a prominent differential expression for *TIMP1, DCN, CXCL8, COL6A1, COL1A2, TGFB1* and *IGFBP7*. (C) The pathway analysis underscores an increase in positive regulation of cell migration, cell-matrix adhesion, focal adhesion, ECM-receptor interaction, positive regulation of endothelial cell migration, vascular branching and negative regulation of apoptosis.

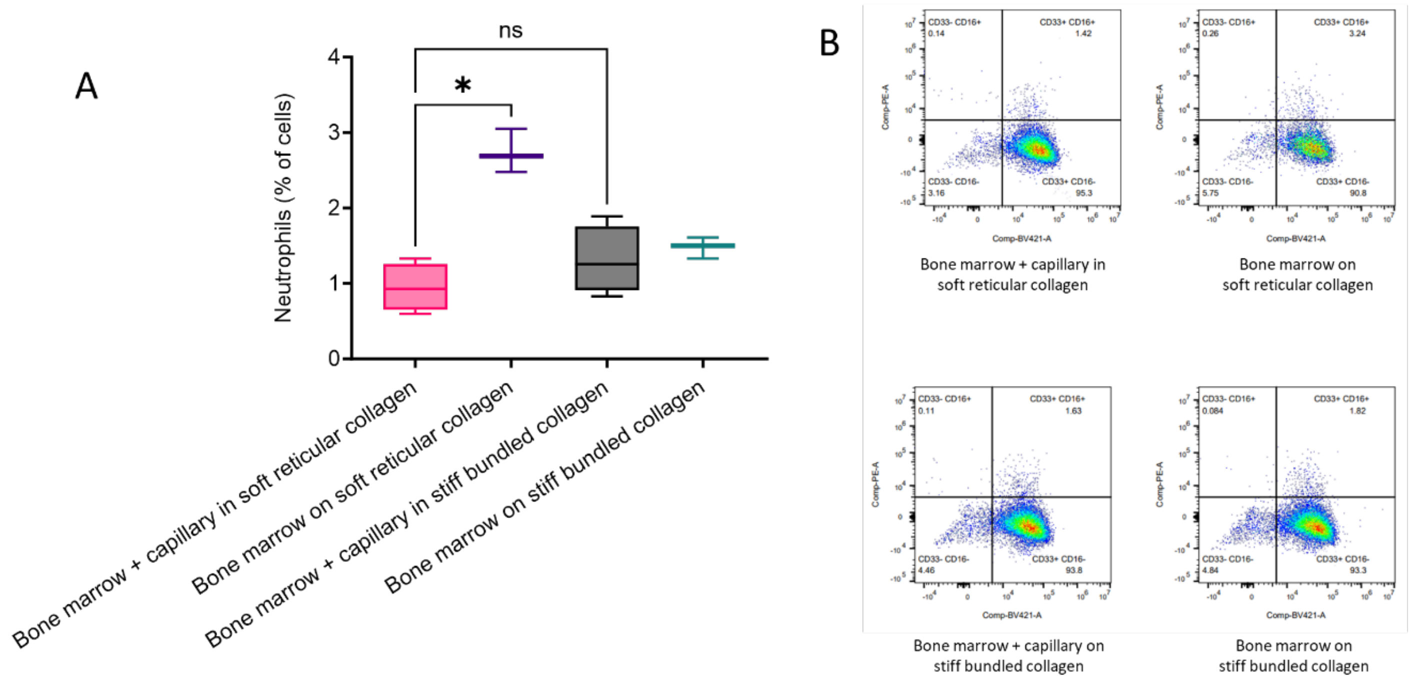

Fig. S18. **Engineered bone marrow differentiation into neutrophilic lineage in proximity to capillaries engineered in either soft reticular or stiff bundled collagen**. (A) Flow cytometry analysis revealed comparable bone marrow cell differentiation into neutrophils regardless of collagen stiffness and microarchitecture. (B) Negative controls were performed by seeding the bone marrow on top of soft or stiff bundled collagen without the vasculature. Bone marrow seeded on top of soft reticular collagen led to an increased percentage of neutrophil differentiation. All experiments were done at least in triplicate. One-way ANOVA post hoc Tukey, *$p<0.05$.

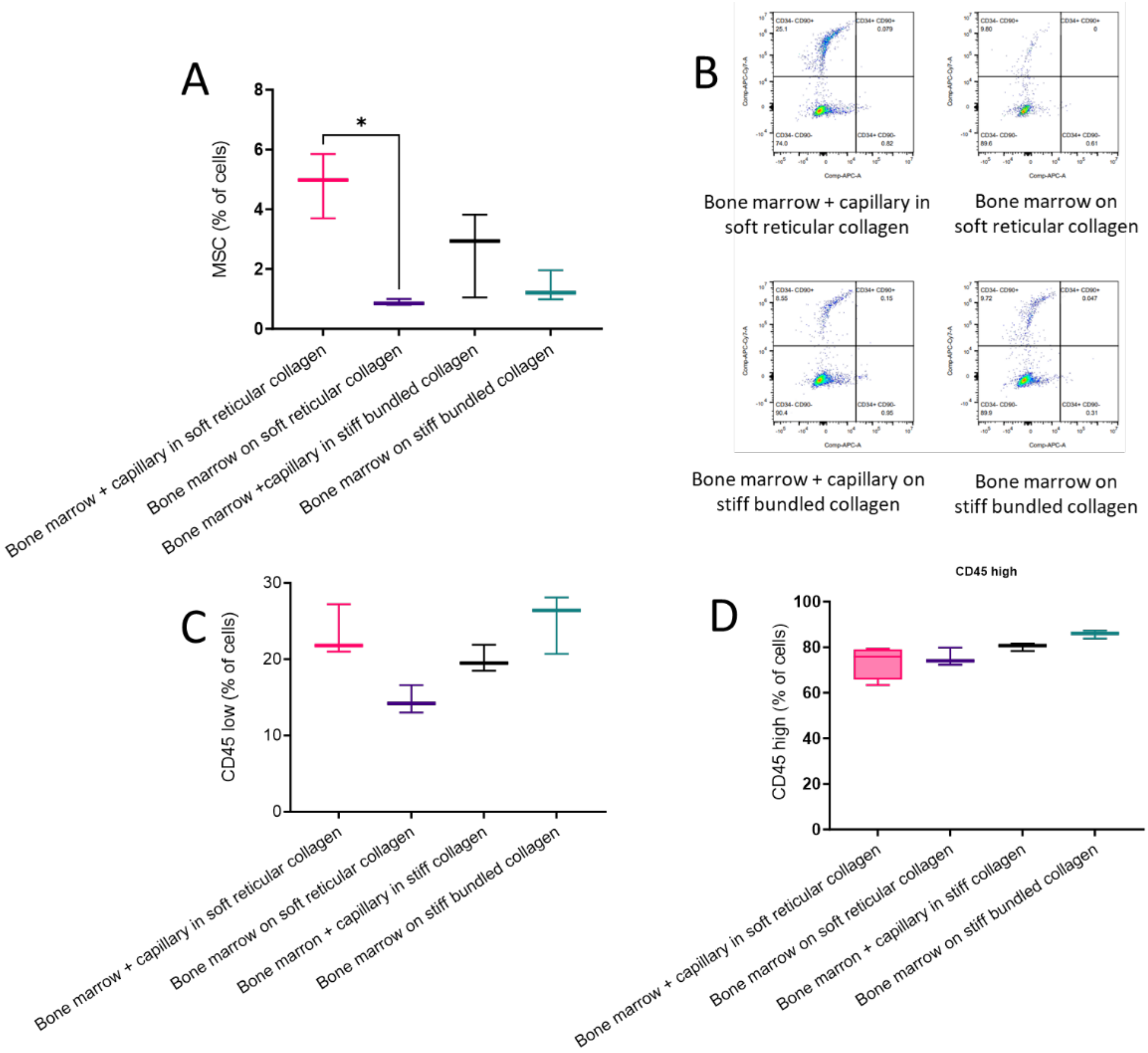

Fig. S19. **Engineered bone marrow differentiation in proximity to healthy (soft) or more fibrotic (stiff) vasculature**. (A, B) Bone marrow seeded on top of healthy/soft collagen resulted in a decreased percentage of hMSC differentiation. (C, D) Flow cytometry analysis revealed comparable bone marrow cell differentiation toward mesenchymal stem cell lineage, as well as the presence of CD45 high or low, indicating consistent outcomes across both vascular conditions. Negative controls were performed by seeding the bone marrow on top of soft or stiff collagen without the vasculature. All experiments were done at least in triplicate. One-way ANOVA post hoc Tukey, $*p<0.05$.

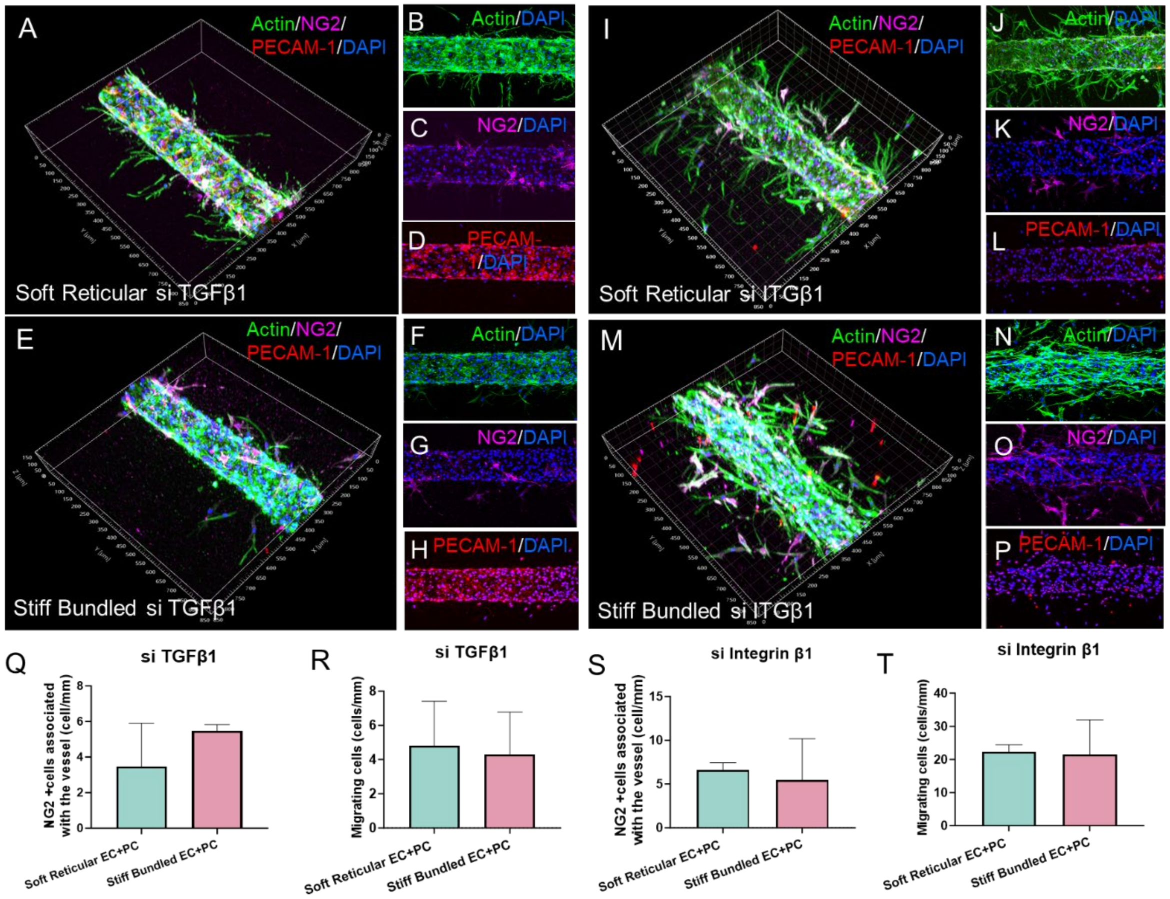

Fig. S20. **Investigating the role of transforming growth factor 1 (TGFβ1) and integrin beta 1 (ITGβ1) in perivascular cell sensing of collagen mechanical properties**. (A-H, Q-R) Silencing *TGFB1* gene normalizes pericyte coverage and migratory cell numbers in fibrotic vasculature, while silencing integrin B1 (*ITGB1*) (I-P, S,T) disrupts healthy vasculature, resembling fibrotic vessel traits with increased migration and reduced pericyte coverage. Note the large increase in Y-axis scaling when quantifying migrating cells in panel T. One-way ANOVA post hoc Tukey, no statistical difference.

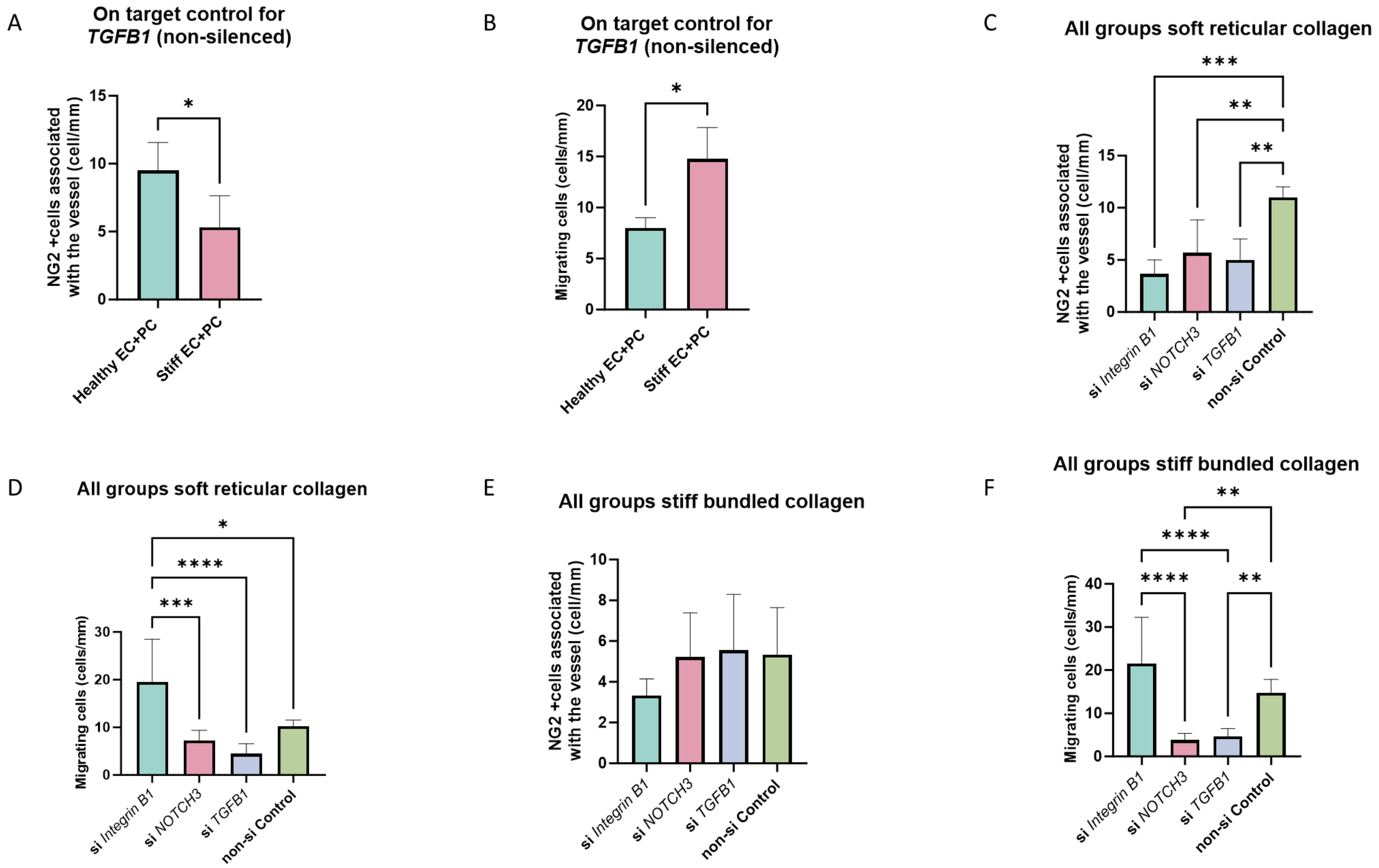

Fig. S21. **Controls for silencing experiments.** (A,B) Non-silenced controls for *TGFB1* experiments. (C) Quantification of NG2+ cells for all silenced genes – *Integrin B1, NOTCH3* and *TGFB1* – in vasculature engineered in soft reticular collagen, showing that after silencing, all groups presented less NG2+ cells than non-silenced groups. (D) Quantification of migrating cells in the vasculature after silencing the genes demonstrating that silencing *Integrin B1* resulted in more cell migration than the control in soft reticular collagen. (E) Silencing the genes in vasculature engineered in the stiff bundled collagen did not alter the number of NG2+ cells. However, (F) migration was decreased after *NOTCH3* and *TGFB1* were silenced. One-way ANOVA post hoc Tukey, * $p<0.05$, ** $p<0.01$, *** $p<0.001$, **** $p<0.0001$.

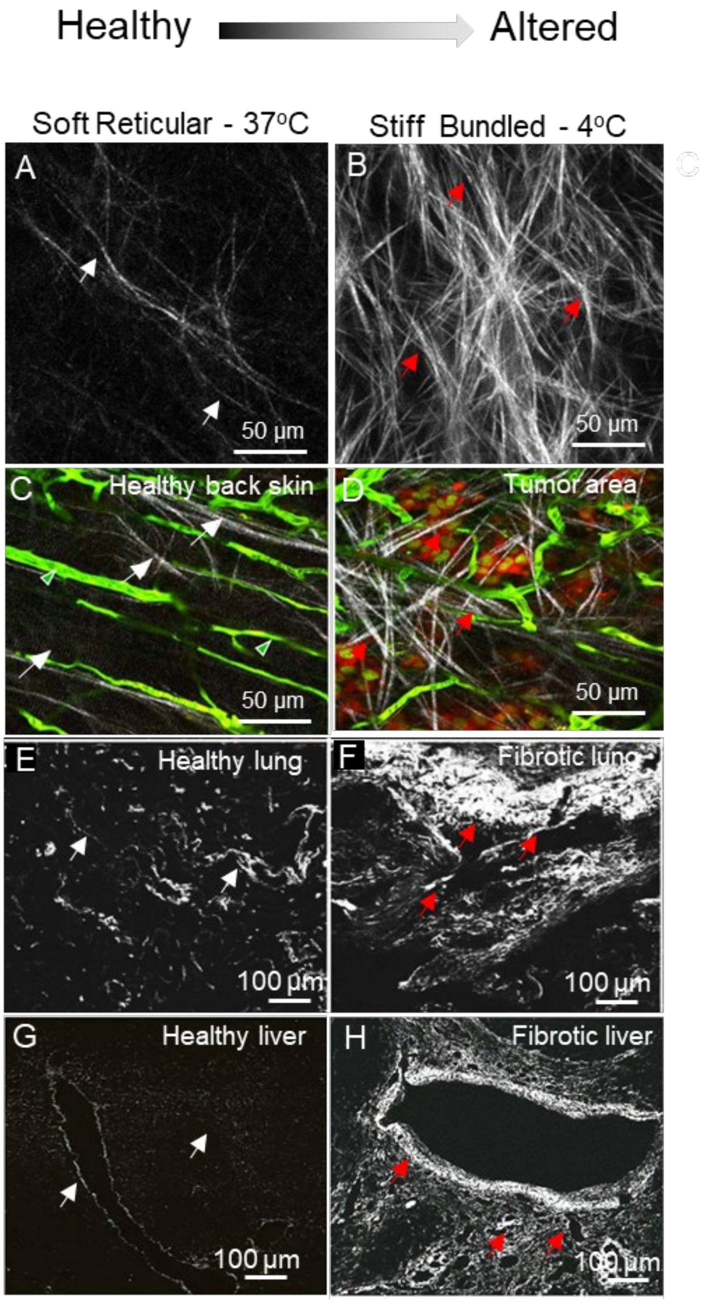

Figure S22: Second harmonic generation (SHG) images of collagen in vitro and in vivo. SHG imaging reveals collagen fibers aligned parallel to the SHG analyzer as white structures, while fibers oriented at different angles are not detected, resulting in a black background. (A) Collagen assembled at 37°C shows thin fibers (white arrows), while (B) collagen assembled at 4°C exhibits thicker fibers with a bundled architecture (red arrows). (C,D) Adapted images from Alexander et al., 2008 show live SHG imaging using a modified skin-fold chamber in mice showing collagen in white, green fluorescence from vessels (FITC green arrow heads) and RFP-expressing fibrosarcoma cells demonstrating that collagen becomes thicker and more bundled in the tumor front, in a pattern similar to images (A,B). (E,F) Adapted images from Kottman et al., 2015, show thin fibers in healthy lung tissue and more abundant, thicker fibers in fibrotic lungs. (G,H) Adaptation from Matsuzaki et al. 2023, comparing liver samples from autopsy cases, dysplaying collagen in healthy livers versus stage 4 fibrotic livers.

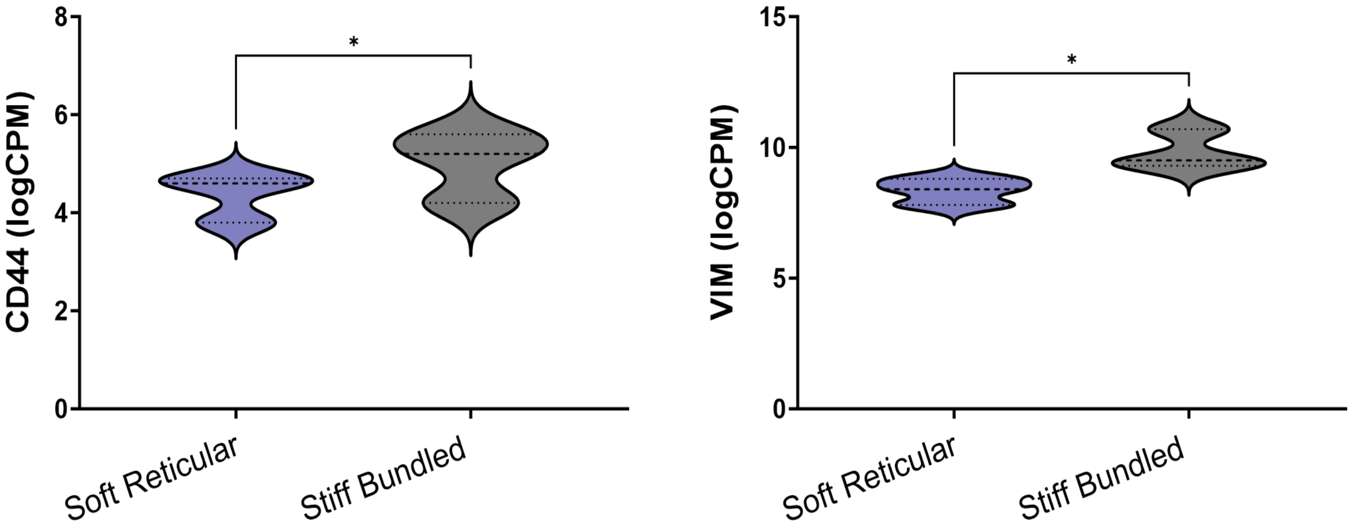

Fig. S23. Gene expression of hMSC markers – Both CD44 and vimentin gene expression is upregulated in the capillaries engineered in the stiff bundled collagen, suggesting that fewer hMSCs undergo differentiation into pericytes while keeping their stemness.

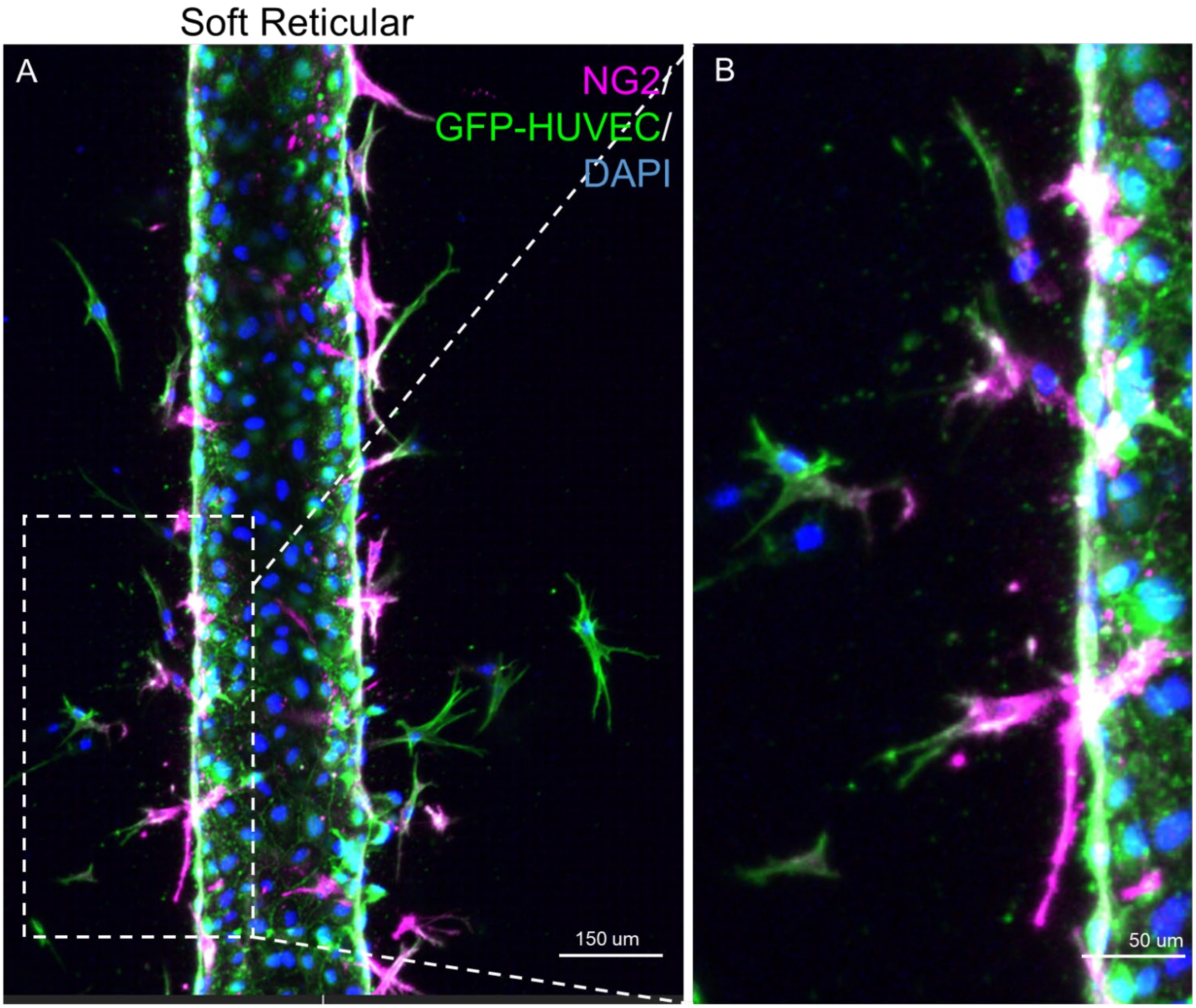

Fig. S24. NG2 expression in perivascular cells relative to proximity to endothelial cells. Image from vasculature engineered in soft reticular collagen. The data indicate a reduction in NG2 expression in perivascular cells that have migrated furthest from the endothelial cells.

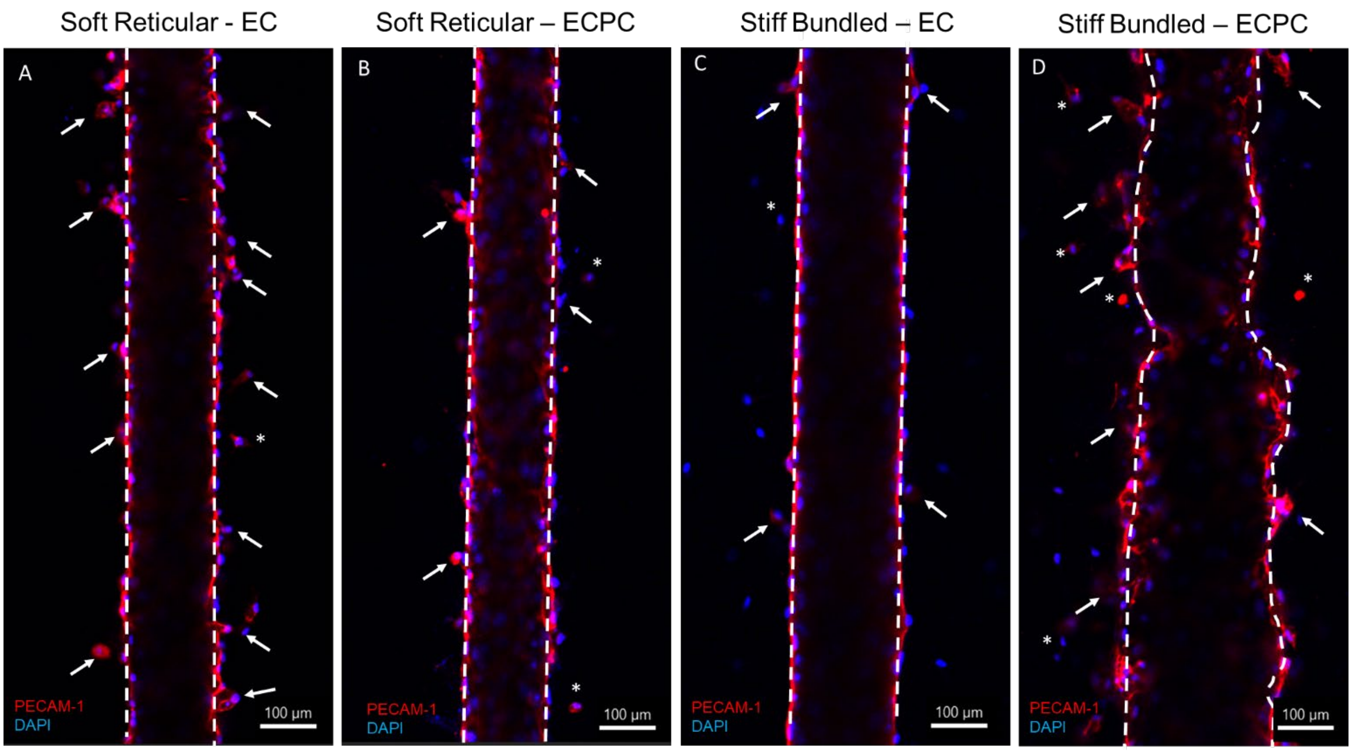

Fig. S25. Representative images of the method used for sprout quantification. For all images, vessels were labelled with PECAM-1 (red) and DAPI (blue). Using the slicing tool in Imaris, longitudinal sections of vessels from each group were imaged, a line was manually traced to delimit the vessel wall and the projections that had tip cells outside the base of the vessel connected to the vessel wall by stalk cells were considered sprouts (arrows). The independent tip cells that were not connected to stalk cells were marked with asterisks (*).

Movie S1. Capillary engineered with perivascular cells in soft reticular collagen akin healthy collagen. Cells were immunostained for actin (green), PECAM-1 (red), NG2 (magenta), DAPI (blue).

Movie S2. Capillary engineered with perivascular cells in stiff bundled collagen akin more fibrotic collagen. Cells were immunostained for actin (green), PECAM-1 (red), NG2 (magenta), DAPI (blue).

Movie S3. Barrier function assay in a healthy capillary engineered within a soft reticular collagen (37ºC).

Movie S4. Barrier function assay in a capillary engineered within an intermediate reticular collagen (21ºC).

Movie S5. Barrier function assay in a capillary engineered within an intermediate bundled collagen (16ºC).

Movie S6. Barrier function assay in a capillary engineered within a fibrotic highly fibrillar collagen (4ºC).

Movie S7. Barrier function assay in a capillary engineered within a highly reticular collagen (37ºC) and perivascular cells with on-target control gene for *NOTCH3* (soft reticular control group).

Movie S8. Barrier function assay in a capillary engineered within a highly reticular collagen (37ºC) and perivascular cells with silenced gene for *NOTCH3* (silenced soft reticular group).

Movie S9. Barrier function assay in a capillary engineered within a stiff bundled collagen (4ºC) and perivascular cells with on-target control gene for *NOTCH3* (stiff bundled control group).

Movie S10. Barrier function assay in a capillary engineered within a stiff bundled (4ºC) collagen and perivascular cells with silenced gene for *NOTCH3* (silenced stiff bundled group).

Dataset S1. Worksheet listing the 770 genes analyzed in the Nanostring PanCancer Progression gene panel.
